# Local Cattle and Badger Populations Affect the Risk of Confirmed Tuberculosis in British Cattle Herds

**DOI:** 10.1371/journal.pone.0018058

**Published:** 2011-03-28

**Authors:** Flavie Vial, W. Thomas Johnston, Christl A. Donnelly

**Affiliations:** 1 Department of Infectious Disease Epidemiology, MRC Centre for Outbreak Analysis and Modelling, School of Public Health, Imperial College London, London, United Kingdom; 2 Department of Health Sciences, University of York, York, United Kingdom; Veterinary Laboratories Agency, United Kingdom

## Abstract

**Background:**

The control of bovine tuberculosis (bTB) remains a priority on the public health agenda in Great Britain, after launching in 1998 the Randomised Badger Culling Trial (RBCT) to evaluate the effectiveness of badger (*Meles meles*) culling as a control strategy. Our study complements previous analyses of the RBCT data (focusing on treatment effects) by presenting analyses of herd-level risks factors associated with the probability of a confirmed bTB breakdown in herds within each treatment: repeated widespread proactive culling, localized reactive culling and no culling (survey-only).

**Methodology/Principal Findings:**

New cases of bTB breakdowns were monitored inside the RBCT areas from the end of the first proactive badger cull to one year after the last proactive cull. The risk of a herd bTB breakdown was modeled using logistic regression and proportional hazard models adjusting for local farm-level risk factors. Inside survey-only and reactive areas, increased numbers of active badger setts and cattle herds within 1500 m of a farm were associated with an increased bTB risk. Inside proactive areas, the number of *M. bovis* positive badgers initially culled within 1500 m of a farm was the strongest predictor of the risk of a confirmed bTB breakdown.

**Conclusions/Significance:**

The use of herd-based models provide insights into how local cattle and badger populations affect the bTB breakdown risks of individual cattle herds in the absence of and in the presence of badger culling. These measures of local bTB risks could be integrated into a risk-based herd testing programme to improve the targeting of interventions aimed at reducing the risks of bTB transmission.

## Introduction

Bovine tuberculosis (bTB) remains an important public health concern worldwide as a result of deficiencies in preventing and/or controlling measures targeting the spread of its causative agent *Mycobacterium bovis* [1], [2]. While the risk posed by *M. bovis* to human health is low in most developed countries, the main causes of concern related to *M. bovis* in industrialized countries are epizootics in domesticated and wild mammal populations [2]. Infection with *M. bovis* remains a significant livestock zoonosis in the European Union where some member states experience a reemergence of the disease despite significant historical efforts to implement eradication plans. In Great Britain, the disease was eliminated from most cattle herds by 1960, with the exception of infection hotspots in southwest England, after the implementation of a herd testing and slaughter policy [3]. However, efforts to completely eradicate bTB in Great Britain have been hampered by the maintenance of *M. bovis* in wildlife host populations, acting as reservoirs of infection, in particular badgers (*Meles meles*) [4]. Since 1979, incidence in British cattle has increased and the infection has become more geographically widespread [5]. Over 7 million cattle were tested for bovine bTB in 2009 and one in ten herds experienced bTB-related movement restrictions during the year [6] as a result of at least one member of the herd failing the tuberculin skin test or showing lesions consistent with bTB during the slaughterhouse inspection – an event known as a “herd breakdown”.

Risk factors associated with bTB have been investigated in case-control studies in Europe and the USA [7], [8], [9], [10], [11], [12], [13], [14]. Historical incidence of bTB was found to be a robust predictor of the rate of future outbreaks in both Irish [15] and British [16] herds, an indication that the source of the disease failed to be eliminated and/or that some factors in those areas make them particularly suitable for the recurrence of infection in cattle. Herd size has repeatedly been identified as one of the major bTB herd-level risk factor [16], [17], [18]. Large herds tend to pasture on larger areas, with higher probabilities of contiguous herds thereby facilitating cattle to cattle spread of *M. bovis* [7]. A comparative case-control study in England between 1995 and 1999 revealed that herd size was a significant predictor of both transient and persistent bTB breakdowns and associated herd size with management-related risk factors such as turnover rates, farm enterprise and feeding [10]. The same study also revealed that farms with higher stocking density showed a significantly reduced risk of a bTB breakdown [10]. Farm size, in terms of number of holdings but not total area farmed, was found to be associated with an increased bTB risk in England beyond any effect of herd size [9]. Cattle housing-type and feeding [9], [10] as well as cattle purchase and movement [9], [19], [20] onto the farm have also been associated with an increased risk of bTB breakdown. With older animals being more likely to have been exposed to *M. bovis* than younger ones [7], dairy cattle, with their longer life expectancy tend to be more at risk of bTB than their beef counterparts [15], [18], [21]. Other differences in terms of management are involved such as higher production stress under intensive management conditions [13] and the twice-daily gathering of cattle during milking which increases the risk of transmission through the respiratory route [22].

*M. bovis* can infect a wide range of wild animals [23], [24]. Brush-tail possums (*Trichosurus vulpecula*) are the primary wildlife reservoir of bovine bTB in New Zealand [25], while white-tail deer (*Odocoileus virginianus*) in Michigan [26], the wood bison in Canada (*Bison bison athabascae*) [27], the buffalo (*Syncerus caffer*) in Southern Africa [28], the wild boar (*Sus scrofa*) in Southern Europe [29], [30] and badgers in Western Europe [4] have become maintenance hosts for *M. bovis*. The Randomised Badger Culling Trial (RBCT) was launched in 1998 to evaluate the effectiveness of badger culling as a control strategy for bTB in Britain [8]. The RBCT involved comparing the incidence of cattle bTB under three experimental treatments — repeated widespread (“proactive”) culling, localized (“reactive”) culling, and no culling (“survey-only”) — each replicated ten times in large (100 km^2^) trial areas recruited as matched sets of three, known as “triplets”. Detailed field surveys in all trial areas for which consent was obtained (see Methods) were undertaken to record the location of badger setts and other field signs of badgers such as latrines and paths. Culling in proactive areas did not start simultaneously in all triplets, with initial proactive culls ranging from December 1998 for triplet B to December 2002 for triplet D. The final proactive cull was completed in late 2005. Many earlier analyses of the RBCT have been published [9], [31], [32], [33], [34], [35], [36], [37], [38], [39], [40], and more details on the RBCT itself can be found in the supplementary information of [36].

In this paper, we present new analyses of spatial herd-level risks factors associated with the probability of bTB breakdowns in herds within the RBCT following the first proactive cull. We examine the extent to which proactive badger culling decreased the bTB risk for the herds involved. We also examine the impact of various local herd-level risk factors within each of the trial group (proactive, survey-only and reactive) to identify the most important bTB breakdown risk factors for herds within the RBCT areas.

## Materials and Methods

### Description of the dataset

The Defra animal health information system (VETNET) provided data on cattle bTB tests and herd breakdowns, distinguishing between “confirmed breakdowns” (incidents in which postmortem examination of slaughtered cattle led to detection of bTB lesions or culture of *M. bovis*) from “unconfirmed breakdowns” (incidents in which one or more cattle reacted to the tuberculin test but infection was not confirmed at postmortem or by culture). Herds with the same County Parish Holding Herd numbers (CPHH: unique herd identifier) which were registered in different treatment groups (n = 14); herds which were archived before the start of the RBCT (n = 22) and herds which showed no evidence of having had a bTB disclosing test during the RBCT (n = 745) were removed from the VETNET records; leaving us with 1306 unique herds recorded in RBCT proactive areas, 1380 unique herds recorded in RBCT survey-only areas and 1320 unique herds recorded in RBCT reactive areas.

Here our analyses were based on the number of confirmed herd breakdowns within treatment groups using information on herd location within trial areas (Table 1). In addition, a survival (or time-to-breakdown) time for each herd was calculated as the time from the end of the initial proactive cull to their first confirmed herd breakdown or to the date of the end of the trial for that triplet or to the date the herd was archived, whichever came first. In the latter case, the time-to-breakdown time was censored. Consent to survey and cull was sought from land owners in all trial areas before random allocation of the treatments and during the course of the trial (Table 2). Following treatment allocation, initial culls were conducted on all land in the proactive areas for which consent was given between 1998 and 2002. These were followed by approximately annual culls until 2005, except during 2001 when culling was suspended during the nationwide epidemic of foot-and-mouth disease. Measures of badger activity before the first proactive cull are described in detail in ref [36].

**Table 1:** Number of cattle herds with and without confirmed bTB breakdowns between the completion of the initial proactive cull and up to one year following the last proactive cull in each of the RBCT triplets.

Triplet	Region	Completion of initial proactive cull	Herds with no bTB breakdown	Herds with ≥1 bTB breakdown
A	Gloucestershire/Herefordshire	Jan-2000	182	114
B	North Cornwall/North Devon	Dec-1998	331	153
C	East Cornwall	Oct-1999	355	166
D	Herefordshire	Dec-2002	177	105
E	North Wiltshire	May-2000	208	108
F	West Cornwall	July-2000	433	107
G	Derby/Staffordshire	Nov-2000	417	131
H	Devon/Somerset	Dec-2000	236	85
I	Gloucestershire	Oct-2002	197	79
J	Devon	Oct-2002	316	106

**Table 2:** The mean number of badger setts identified during the initial survey and badgers culled during the first proactive cull on and around farms for all three treatment groups.

		Mean number of badger setts identified during the initial survey		Mean number of badgers culled during the first proactive cull (*M. bovis* +)	
	% of landholders refusing access 1 (total)	on the farms	within a 1500 m buffer	on the farms	within a 1500 m buffer
Survey-only	12% (1380)	1.90	26.76	NA	NA
Proactive	11% (1306)	2.04	29.97	1.95 (0.66)	27.27 (2.87)
Reactive	10% (1320)	2.25	28.95	NA	NA

1 Some landholders did not consent to survey and/or cull badgers on their land.

#### The variables

On the basis of details recorded in the RBCT database, farms were categorized into one of three enterprise types: beef, dairy and other (a composite category including calf rearers, dealers, exempt finishing units, heifer rearers, house cows, mixed herds and stores). The median herd size was 72 animals (mean  = 102, standard error  = 1.7) [Supplementary Information S1]. The historic incidence of cattle bTB (number of confirmed herd breakdowns) was calculated for each trial area, for the three-year period before the initial proactive cull, except in triplets D, I and J where it was calculated for the three years prior to the start of the 2001 foot-and-mouth disease epidemic (median  = 25 confirmed breakdowns, mean  = 25.37, s.e. = 0.12) [Supplementary Information S1]. The median number of baseline herds in the triplets (number of herds recorded for that triplet at the time of the initial badger cull) was 124 (mean  = 133.70, s.e. = 0.74). Some farms operate on more than one land parcel (defined as a discrete piece of land discontinuous with neighbouring land). Farm area was then computed as the combined area of all land parcels belonging to a particular farm. Most farms operated from two land parcels (median  = 2, mean  = 2.14, s.e. = 0.03, max  = 16) and median farm area was estimated at 0.50 km^2^ (mean  = 0.69, s.e. = 0.012).

Data on the number/density of badgers culled, the number/density of *M. bovis* positive (+) badgers culled, the number/density of active badger setts and the number/density of neighbouring cattle herds within 500, 1000 and 1500 m of all the land parcels belonging to a farm were extracted from the RBCT geodatabase (ArcGIS version 9, ESRI) [Supplementary Information S1]. On land parcels for which consent to survey and/or cull was given, distinct badger and sett-related variables could be produced to reflect numbers/densities on the land parcels themselves versus numbers/densities on the buffer surrounding the parcels. When consent to cull and/or survey was not obtained (Table 2), trapping along the boundaries of the parcels for which consent was refused allowed staff to catch a proportion of the badgers residing in the no-access farm. The area of the farm and the buffer was thus used when calculating the density of badgers trapped (on both the parcels and the buffer) but not when calculating sett density (which could only be estimated inside the surveyed buffer)[Supplementary Information S1].

### Statistical analyses

The significance of the following local farm-level risk factors were assessed (herd type, herd size, farm area within the triplet, the number of baseline herds, historic incidence within the trial areas, and the number of premises operated by the farm in the first instance) and subsequent models were adjusted accordingly. A distinction was made for badger-related and sett-related variables between the number of badgers culled or setts recorded on the farm's land parcels and those on the buffer surrounding the farm. This distinction was only retained in the multivariable models if significant. The badger-related, sett-related and herd-related variables which demonstrated the most significant univariable associations with the risk of confirmed herd breakdowns were retained for multivariable model building. All models adjusted either for herd type, herd size, farm area (models A); for herd type, herd size, farm area and historic bTB incidence (models B) or for herd type, herd size, farm area and triplet [Supplementary Information S1]. Models were constructed by backward elimination, starting with a full model with quadratic terms for each non-categorical variable. Variables were eliminated on the basis of their significance in the model as well as their contribution to the variation in the data by means of an analysis of variance using a F-test (for the logistic regressions) or a likelihood ratio test (LRT) in which twice the difference in log-likelihoods was compared to a Chi-square (χ^2^) distribution otherwise. An F-test was chosen for the logistic regressions as a result of overdispersion in our data. To minimize bias in the covariates, 0.5 was added before log-transforming all non-categorical variables.

#### Probability of confirmed bTB herd breakdown

Using the herds that did not experience any bTB breakdown during the period under study as controls, we used logistic regression to compare the probability of one or more confirmed herd bTB breakdowns for each herd recorded inside trial areas subjected to the proactive and survey-only treatments. In addition, we used logistic regression to model the probability of one or more confirmed bTB herd breakdowns during the period under study for each herd within a particular treatment (proactive, reactive or survey-only). Variables were individually screened using logistic regression controlling for local farm-level risks [Supplementary Information S1]. P-values were adjusted for overdispersion, when present, by using an inflation factor equal to the square root of the model deviance divided by the degrees of freedom. An assessment of the goodness-of-fit was obtained by examining the models' residuals.

#### Time to first confirmed bTB herd breakdown

Analysis of these data was undertaken using proportional hazards (PH) models, comparing the time to the first confirmed bTB breakdown for herds recorded inside trial areas subjected to the proactive and survey-only treatments. PH models were also used to predict the time to first confirmed breakdown for herds within a particular treatment group (proactive, reactive or survey-only). The badger-related, sett-related and herd-related variables which demonstrated the most significant univariable associations with time to first confirmed bTB herd breakdown were then retained for multivariable model building controlling for local farm-level risks [Supplementary Information S1]. The proportional-hazards assumption was tested for each covariate, by correlating the scaled Schoenfeld residuals with the Kaplan-Meier estimate of the survival function [41]. Other model diagnostics included checking the martingale residuals to detect non-linearity.

## Results

Data from 4006 herds were available for the analysis: 343 out of 1306 proactive herds, 408 out of 1380 survey-only herds and 403 out of 1320 reactive herds experienced a confirmed bTB breakdown between the completion of the initial proactive badger cull within their triplet and one year following their final proactive cull.

### Probability of confirmed bTB herd breakdown

Overall, when comparing the probabilities of confirmed herd bTB breakdowns during the period under study between proactive and survey-only herds, we found that the best model included effects of triplet (p = 0.04), herd type, herd size, farm area and the historic bTB incidence for that trial area. The analyses of variance showed that the number of land parcels belonging to the farm (p = 0.79) and the number of baseline herds were not significant (p = 0.45). Culling treatment (p = 0.07) was also non-significant although there was a trend for reduced bTB risks among herds in proactively culled areas (OR: 1.19, 95% CI: 0.98–1.44).

Herds categorized under the “other” enterprise type, had a similar risk of bTB breakdown to that of beef herds (p = 0.75), so both types were then merged to create a “non-dairy” group. Dairy herds showed a significantly higher risk of bTB breakdown (p = 0.014) compared to non-dairy herds (OR: 1.30, 95% CI: 1.09–1.75). Larger herds (p<0.001, OR: 1.20, 95% CI: 1.13, 1.26) and bigger farms presented an increased risk of bTB breakdown (p<0.001, OR: 1.30, 95% CI: 1.22–1.39). The odds ratio here are interpreted as a doubling of the herd size or of farm area resulting in a 20% and 30% increase, respectively, in the odds of a bTB breakdown. As expected, historic bTB incidence for trial area of the herd was also a significant predictor (p<0.001) of its probability of experiencing a bTB breakdown after the initial proactive cull (OR: 2.25, 95% CI: 1.96, 2.54 corresponding to a doubling of the historic incidence).

#### Within survey-only areas

The number of active badger setts (both on the land parcels and outside but within 500 m) as well as the number of cattle herds within 500 m of all land parcels were the best individual predictors of the probability of a confirmed bTB breakdown for survey-only herds during the period under study [Supplementary Information S1]. Both variables remained significant predictors in the multivariable logistic model (Table 3). An increase in the number of active setts and cattle herds within the 500 m wide buffer surrounding the farm's land parcels resulted in an increased bTB risk (Table 3). Both risk factors were consistent across the 1000 m and 1500 m wide buffer [SSupplementary Information S1].

**Table 3:** Multivariable models of the probability of RBCT herds experiencing a confirmed bTB breakdown during the period under study.

	Survey-only		Proactive		Reactive	
	Model A 1	Model B	Model A	Model B	Model A	Model B
Number of *M. bovis* + badgers culled on the land parcels 2	NA	NA	----	----	NA	NA
Number of *M. bovis* + badgers culled outside but within 500 m	NA	NA	p<0.001	p = 0.002	NA	NA
			OR: 1.27	OR: 1.22		
			(1.15–1.39)	(1.10–1.35)		
Number of active setts on the land parcels 3	----	----	----	----	NA	NA
Number of active setts outside but within 500 m	p = 0.003	p = 0.02	----	----	NA	NA
	OR: 1.13	OR: 1.14				
	(1.02–1.24)	(1.03–1.25)				
Density (/km^2^) of active setts on the land parcels	NA	NA	NA	NA	----	----
Density (/km^2^) of active setts outside but within 500 m	NA	NA	NA	NA	----	----
Number of cattle herds tested 4	p = 0.001	p = 0.004	NA	NA	p<0.001	p<0.001
	OR: 1.44	OR: 1.38			OR: 1.69	OR: 1.75
	(1.22–1.66)	(1.16–1.61)			(1.49–1.89)	(1.55–1.96)
Density (/km^2^) of cattle herds tested	NA	NA	----	----	NA	NA
Herd type [DAIRY]	p = 0.47	p = 0.25	p = 0.03	p = 0.04	p = 0.13	p = 0.15
	OR: 1.13	OR: 1.22	OR: 1.45	OR: 1.42	OR: 0.76	OR: 0.77
	(0.81–1.57)	(0.87–1.71)	(1.03–1.69)	(1.01–2.00)	(0.53–1.08)	(0.54–1.10)
Herd size	p<0.001	p<0.001	p = 0.003	P = 0.002	p<0.001	p<0.001
	OR: 1.21	OR: 1.21	OR: 1.18	OR: 1.19	OR: 1.25	OR: 1.26
	(1.11–1.31)	(1.11–1.32)	(1.07–1.28)	(1.08–1.30)	(1.15–1.36)	(1.15–1.37)
Farm area	p = 0.002	p<0.001	p<0.001	p<0.001	p = 0.007	p = 0.01
	OR: 1.22	OR: 1.25	OR: 1.30	OR: 1.33	OR: 1.19	OR: 1.18
	(1.09–1.35)	(1.12–1.38)	(1.17–1.43)	(1.20–1.46)	(1.07–1.32)	(1.04–1.31)
bTB historic incidence within trial area	NA	p<0.001	NA	p<0.001	NA	p = 0.006
		OR: 2.16		OR: 2.51		OR: 1.62
		(1.74–2.58)		(2.14–2.88)		(1.27–1.96)

Odds ratios (OR) are quoted with their corresponding 95% confidence interval, and for covariates correspond to the change in the risk of a confirmed bTB breakdown following a doubling of the value of the covariate.

The --- means that an individual predictor was not significant and removed from the model, while NA corresponds to variables that were not included following the screening process.

1 Models are adjusted for herd size, herd type and farm area (model A); herd size, herd type, farm area and bTB historic incidence within the trial area (model B).

2 Relate to the badgers culled during the initial proactive cull.

3 Relate to the badger setts identified during the initial survey.

4 Relate to herds tested for bTB during the one year prior to the start of the initial proactive cull.

#### Within proactive areas

The number of *M. bovis* positive culled badgers, the number of active badger setts (both on the land parcels and outside but within 500 m) as well as the density of cattle herds within 500 m of the land parcels were the best individual predictors of the probability of a confirmed bTB breakdown for proactive herds during the period under study [Supplementary Information S1]. The number of *M. bovis* positive badgers that were culled outside but within 500 m of the land parcels belonging to a farm remained the only significant predictor in the multivariable logistic model. An increase in the number of *M. bovis* positive badgers culled within the 500 m wide buffer surrounding the farm's land parcels resulted in an increased bTB risk (Table 3). The risk factor was consistent across the 1000 m and 1500 m wide buffer [Supplementary Information S1]. Although non-significant, the number of active badger setts (p = 0.50, OR: 1.05, 95% CI: 0.92–1.17 corresponding to a doubling in the number of setts) and the number of cattle herds (p = 0.50, OR: 1.08, 95% CI: 0.86–1.29 corresponding to a doubling in the number of herds) outside but within 500 m of the lands parcels (risk factors indentified for the survey herds) resulted in a marginal increase in bTB risk (model B). Thus, these effects were in the same direction as those observed in survey-only areas.

#### Within reactive areas

The density of active badger setts (both on the land parcels and outside but within 500 m) as well as the number of cattle herds within 500 m of the land parcels were the best individual predictors of the probability of a confirmed bTB breakdown for reactive herds [Supplementary Information S1]. The number of cattle herds inside a 500 m wide buffer surrounding all land parcels belonging to a farm remained the only significant predictor in the multivariable logistic model of the probability of a confirmed bTB herd breakdown. An increase in the number of cattle herds within the 500 m wide buffer surrounding the farm's land parcels resulted in an increased bTB risk (Table 3). This risk factor was consistent across the 1000 m and 1500 m wide buffer [Supplementary Information S1]. Although non-significant, the number of active badger setts (p = 0.67, OR: 1.02, 95% CI: 0.91–1.14 corresponding to a doubling in the number of setts) outside but within 500 m of the lands parcels (risk factor indentified for the survey herds) resulted in a marginal increase in bTB risk (model B). Thus, this effect was in the same direction as those observed in survey-only areas.

#### Time to first confirmed bTB herd breakdown

**Figure 1:**
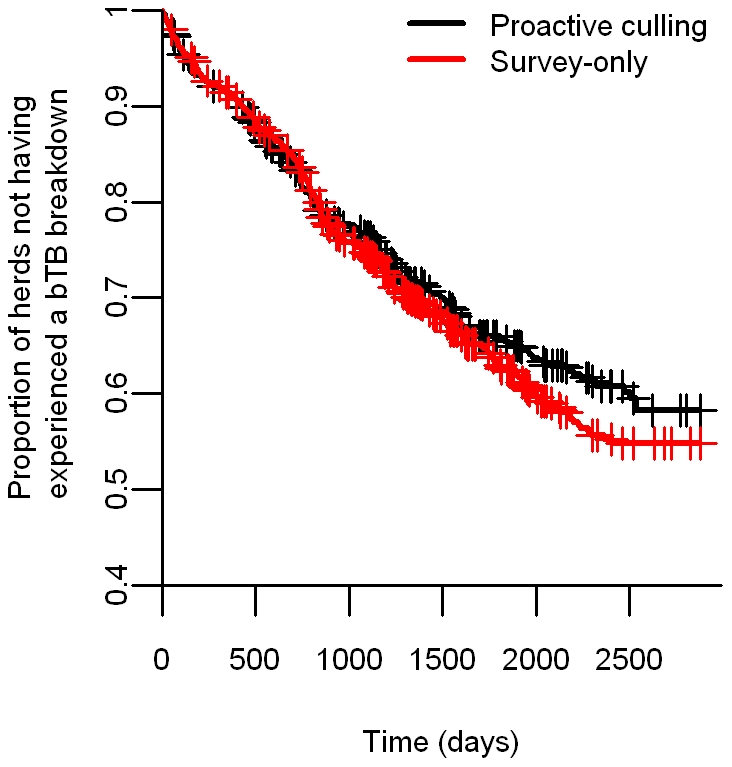
Effect of badger culling on time to first confirmed bTB herd breakdown. The Kaplan-Meier survival curves represent the proportion of proactive and survey-only herds not having experienced a confirmed bTB breakdown as a function of the number of days since the initial proactive cull (the Kaplan-Meier estimator is not adjusted for any other variable). The effect of proactive badger culling on the time to first breakdown is not significant.

Overall, when comparing the time to the first confirmed herd bTB breakdown between proactive and survey-only herds during the period under study, we found that the best model included effects of farm area, herd type, herd size, triplet and the historic bTB incidence within the trial area. LRT showed that the number of land parcels belonging to the farm (p = 0.90), and the number of baseline herds (p = 0.44) were not significant and removed from the model. Culling treatment (p = 0.08) was also non-significant although there was a trend for reduced bTB risks among herds in proactively culled areas (HR: 1.11, 95% CI: 0.99–1.22) (Figure 1). The variable “farm area” showed some evidence of non-proportional hazard (p = 0.04). To resolve this issue, we transformed the variable into a factor with two levels [small farms (area < median farm area) and large farms (area ≥ median farm area]. We found that such procedure had little effect on the non-proportional hazard (p = 0.06), and decided to retain “farm area” as a covariate as none of the other model diagnostics revealed violations of PH assumptions.

**Figure 2:**
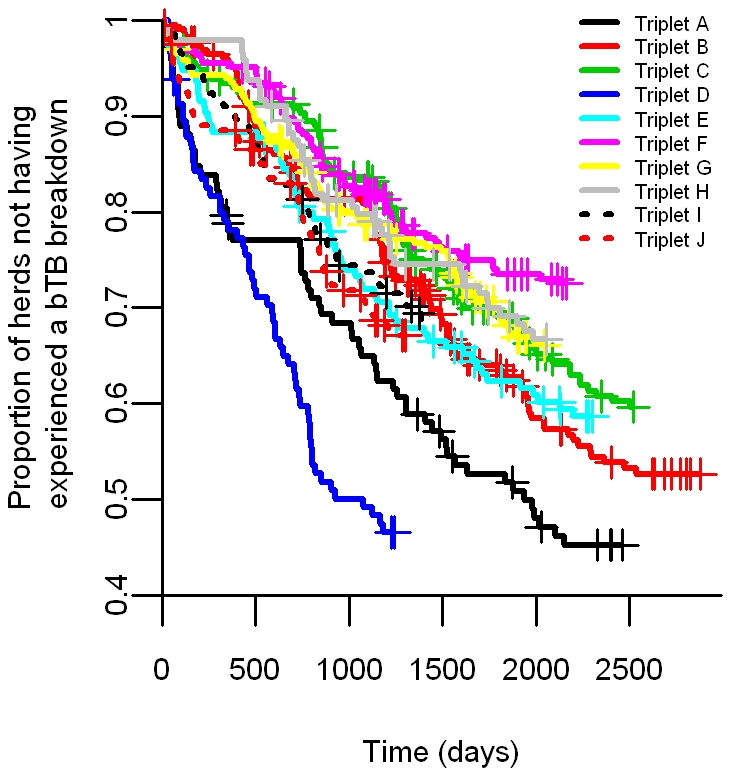
Time to first confirmed bTB herd breakdown for each triplet. The Kaplan-Meier survival curves represent the proportion of herds not having experienced a confirmed bTB breakdown as a function of the number of days since the initial proactive cull for each triplet (the Kaplan-Meier estimator is not adjusted for any other variable). Triplet D had a significantly higher risk of bTB breakdown than all other triplets.

Dairy herds showed a significantly higher risk of bTB breakdown (p = 0.001) compared to non-dairy herds (HR: 1.33, 95% CI: 1.12–1.58). Larger herds (p<0.001, HR: 1.13, 95% CI: 1.08, 1.19) and larger farms (p<0.001, HR: 1.26, 95% CI: 1.19, 1.33) presented an increased risk of bTB breakdown. The hazard ratios here are interpreted as a doubling of the herd size or of farm area resulting in a 13% and 26% increase, respectively, in the hazard of a bTB breakdown. The triplet of the herd (p<0.001), and the historic bTB incidence for the trial area (p<0.001, HR: 2.27, 95% CI: 2.01, 2.52 corresponding to a doubling in historic incidence), were significant predictors of the herd's time to a confirmed bTB breakdown in the period under study. A Tukey's honest significance test revealed that triplet D, the last to receive proactive culling, had a significantly higher risk of bTB breakdown than all other triplets (Figure 2).

#### Within survey-only areas

The number of active badger setts (both on the land parcels and outside but within 500 m) as well as the number of cattle herds within 500 m of all land parcels were the best individual predictors of the time to first confirmed bTB breakdown for survey-only herds [Supplementary Information S1]. Both variables remained significant predictors in the multivariable PH model. An increase in the number of active setts and cattle herds within the 500 m wide buffer surrounding the farm's land parcels resulted in an increased bTB risk (Table 4). Both risk factors were consistent across the 1000 m and 1500 m wide buffer [Supplementary Information S1].

**Table 4:** Multivariable models of the time to first confirmed bTB breakdown for RBCT herds during the period under study.

	Survey-only		Proactive		Reactive	
	Model A ^1^	Model B	Model A	Model B	Model A	Model B
Number of *M. bovis* + badgers culled on the land parcels ^2^	NA	NA	----	----	NA	NA
Number of *M. bovis* + badgers culled outside but within 500 m	NA	NA	p<0.001	p<0.001	NA	NA
			HR: 1.29	HR: 1.25		
			(1.21–1.38)	(1.17–1.34)		
Number of active setts on the land parcels ^3^	----	----	----	----	----	----
Number of active setts outside but within 500 m	p = 0.04	p = 0.03	----	----	p = 0.03	p = 0.04
	HR: 1.09	HR: 1.09			HR: 1.10	HR: 1.09
	(1.01–1.16)	(1.01–1.17)			(1.01–1.18)	(1.01–1.18)
Density (/km^2^) of active setts on the land parcels	NA	NA	NA	NA	----	----
Density (/km^2^) of active setts outside but within 500 m	NA	NA	NA	NA	----	----
Number of cattle herds tested ^4^	p = 0.002	p = 0.01	NA	NA	p<0.001	P<0.001
	HR: 1.30	HR: 1.26			HR: 1.29	HR:1 .32
	(1.14–1.47)	(1.09–1.42)			(1.15–1.44)	(1.18–1.46)
Density (/km^2^) of cattle herds tested	NA	NA	----	----	NA	NA
Herd type [DAIRY]	p = 0.31	p = 0.20	P = 0.02	p = 0.03	p = 0.15	p = 0.17
	HR: 1.13	HR: 1.17	HR: 1.35	HR: 1.33	HR: 0.83	HR: 0.84
	(0.89–1.44)	(0.91–1.48)	(1.05–1.73)	(1.22–1.57)	(0.65–1.07)	(0.65–1.08)
Herd size	p = 0.02	p = 0.02	p = 0.04	p = 0.04	p = 0.001	p<0.001
	HR: 1.10	HR: 1.11	HR: 1.10	HR: 1.09	HR: 1.15	HR: 1.16
	(1.02–1.19)	(1.02–1.19)	(1.01–1.19)	(1.01–1.18)	(1.06–1.24)	(1.07–1.24)
Farm area	p<0.001	p<0.001	p<0.001	p<0.001	p = 0.02	p = 0.03
	HR: 1.21	HR: 1.22	HR: 1.24	HR: 1.26	HR: 1.13	HR: 1.12
	(1.11–1.30)	(1.12–1.32)	(1.14–1.34)	(1.16–1.36)	(1.03–1.24)	(1.02–1.23)
bTB historic incidence within trial area	NA	p = 0.01	NA	p<0.001	NA	p<0.001
		HR: 1.57		HR: 1.87		HR: 1.69
		(1.25–1.89)		(1.59–2.14)		(1.43–1.95)

Hazard ratios (HR) are quoted with their corresponding 95% confidence interval, and for covariates correspond to the change in the risk of a confirmed bTB breakdown following a doubling of the value of the covariate.

Refer to other footnotes from Table 3.

#### Within proactive areas

The number of *M. bovis* positive culled badgers, the number of active badger setts (both on the land parcels and outside but within 500 m) as well as the density of cattle herds within x meters of the land parcels were the best individual predictors of the time to first confirmed bTB breakdown for proactive herds during the period under study [Supplementary Information S1].The number of *M. bovis* positive badgers that were culled outside but within 500 m of the land parcels belonging to a farm remained the only significant predictor in the multivariable PH model. An increase in the number of *M. bovis* positive badgers culled within the 500 m buffer surrounding the farm's land parcels resulted in an increased bTB risk (Table 4). The risk factor was consistent across the 1000 m and 1500 m wide buffer [see Supplementary Information]. Although non-significant, the hazard ratios (model B) corresponding to the number of active badger setts (p = 0.53, OR: 1.03, 95% CI: 0.94–1.12) and the number of cattle herds (p = 0.65, OR: 0.97, 95% CI: 0.82–1.11) outside but within 500 m of the lands parcels are concordant with the ones derived from herds within survey-only areas.

#### Within reactive areas

The number of active badger setts (both on the land parcels and outside but within 500 m) as well as the number of cattle herds within 500 m of the land parcels were the best individual predictors of the time to first confirmed bTB breakdown for reactive herds [Supplementary Information S1]. Both variables remained significant predictors of the time to first confirmed bTB herd breakdown. An increase in the number of cattle herds and active setts within the 500 m buffer surrounding the farm's land parcels resulted in an increased bTB risk (Table 4). The risk factor associated with the number of cattle herds was consistent whether the buffer was 1000 m or 1500 m wide [Supplementary Information S1] while the number of active badger setts acted as a non-significant bTB risk factor on land over 500 m outside the farm.

## Discussion

### Local herd risk factors

A number of local herd-level risk factors have been identified inside all three treatment groups of the RBCT by the present analyses. Some of these risk factors had also been described for herds outside the RBCT area. Dairy herds were found to be more at risk of a confirmed bTB breakdown. Animals in dairy herds tend to have a longer life expectancy, and thus a longer exposure to bTB and increased risk of breakdown [23], than beef cattle that are slaughtered at a young age. Unlike beef farms that use a variety of breeds and crossbred animals, dairy farms in the UK predominantly use one breed of cattle (Ivan Morrison pers. comm.). A breed-related difference in susceptibility may ensue [42] although it is difficult to disentangle its potential effects from higher production stress under more intensive management conditions for dairy cattle for example [43]. Interestingly, dairy herds within the RBCT tended to be much larger than other enterprise types [Supplementary Information S1], another risk factor identified in the present study. Large herds tend to pasture on larger areas, with correspondingly higher numbers of contiguous herds and potential contact with more badgers (if badger densities were constant on all sizes of pastures) thereby facilitating cattle to cattle [7] and badger to cattle spread of *M. bovis*, respectively. Alternatively, large herd size may be associated with management practices that increase the risk of *M. bovis* transmission. Indeed, we found that herd size was positively correlated with the number of cattle movements onto the farms [Supplementary Information S1]. The arrival of an infected animal in a bTB-free herd is one of the major risk factors for herd breakdowns, as suggested by studies carried out in the UK, USA and Italy [9], [17], [19], [44]. Another important consideration relates to the difficulty of clearing bTB from large herds by test and slaughter [45], rendering large herds more at risk of recurrent infections.

Our findings regarding the risk posed by farm area on bTB herd breakdowns were opposite to the ones described by Johnston and colleagues [9]. Total farm area, and not the number of land parcels the farm was operated on, was associated with an increased bTB risk. Larger farms, regardless of the number of land parcels, may include more active badger setts or more contiguous herds, both risk factors identified in this study. The number/density of cattle herds within 500/1000/1500 m of a farm was a significant predictor of herd breakdowns in survey-only and reactive areas. A recent study in Belgium, a country lacking a significant wildlife reservoir for bTB, showed that the larger the livestock population in an area, the higher the probability of close contacts, and bTB transmission, between them [45]. The movement and trading of animals from high bTB risk herds has been found to contribute to both the local and long-distance geographic spreading of the disease [20], [46]. We found that farm area was positively correlated with the number of cattle movements onto the farm [Supplementary Information S1]. Larger farms purchased more animals, suggesting a higher probability of introducing the disease into their herd. The retention of historic bTB incidence in the multivariable models suggest that this risk factor is important in determining whether herds in a parish group are likely to experience a bTB breakdown in a particular year. Herd breakdowns tend to be recurrent [5] possibly as a result of the failure to clear the source of the disease, especially from larger herds, by test and slaughter [45]. Subsequent breakdowns could therefore arise from undetected (tuberculin-negative) infected animals. This factor is probably exacerbated for dairy herds whose turnover is less important than stores or beef enterprises. Other permanent factors (such as the presence of badgers and/or contiguous herds) may make these areas particularly prone to bTB reemergence.

The analyses of RBCT cattle incidence data using individual-herd-based models also provide insights into how local cattle herds and local badger populations affect the breakdown risks on individual cattle herds in survey-only areas (unculled areas). The presence of badgers (measured here as the number of active badger setts) was associated with an increase in bTB risk, even after adjusting for local farm-level risk factors. The higher the number of badger setts identified within 1500 m of the land parcels, the higher the probability of at least one confirmed bTB breakdown for the corresponding herd, a pattern that has also been observed in Northern Ireland [12] and the Republic of Ireland [47]. Similarly, the number of herds within 1500 m of a farm was a very significant predictor of both the probability and the time to the first bTB breakdown for that herd. The larger the cattle population surrounding a farm, the higher the number of contiguous herds that are likely to have had experienced a confirmed bTB breakdown in the past. A case-control study in Northern Ireland showed that the odds of a bTB breakdown are increased by more than two-fold if a herd has a contiguous neighbour which has experienced a confirmed bTB within the last three years [12], with a similar pattern once again observed in the Republic of Ireland [7].

### Effects of badger culling on the risk of bTB herd breakdowns

Previous studies have demonstrated that the experimental reduction of badger density by culling over large (≥100 km^2^) tracts of land lowers the incidence of bTB inside proactively culled areas [36], [48] but increases the incidence on land outside but within 2 km of the area culled [36], [40]. Proactive culling has been demonstrated to reduce local densities of badgers [32], and subsequently cattle to badger contact, with benefits of culling still apparent five years after the last proactive cull [49]. Although a log-linear analysis remains the most robust approach to investigate treatment effects inside the RBCT [36], we find non-significant differences in the probability of (p = 0.07) and the time to (p = 0.008) a confirmed bTB herd breakdown between herds in proactive and survey-only areas during the study period, a finding consistent with previous analyses. There is a non-significant trend for herds in survey-only areas to be 19% more likely to experience a confirmed bTB breakdown than herds in areas that were proactively culled.

More importantly, our study complements previous analyses of the RBCT data by focusing on variation in bTB risk at the herd-level within trial areas (the unit of randomization). The number of culled badgers that tested positive for *M. bovis* inside the buffer surrounding the farm during the initial proactive cull remains a significant predictor of both the probability of experiencing and the time to a confirmed bTB breakdown for herds within the proactive area after the end of the initial cull. Such associations may be indicative of an underlying bTB risk for those herds which has not been eliminated by the proactive badger culling (for example higher bTB prevalence). Our findings suggest that infection in cattle and badgers are linked, and are supported by a previous study which concluded that a high degree of similarity in the *M. bovis* strain types isolated from cattle and associated badgers existed in England [33].

Reactive badger culling caused an increase in bTB incidence recorded in reactive areas [50], likely as a result of expanded badger movement patterns and increased intraspecific transmission following the cull [31], [33]. In this study, we attempted to relate the herd-probability of a bTB breakdown to measures of badger presence measured prior to the start of the reactive culling. We found that the presence of badger setts (outside but within 500/1000 or 1500 m of the land parcels) is a significant predictor of the time to the first bTB breakdown (although this finding is not consistent across all analyses performed - Supplementary Information S1) but was not associated with the probability of a herd experiencing at least one confirmed bTB breakdown.

In conclusion, our findings confirm that proactive culling of badgers, whilst in operation, reduces the individual-herd probability of experiencing a herd bTB breakdown. Increased numbers of badgers carrying *M. bovis* and increased numbers of active badger setts significantly increased the probability of a breakdown for herds in proactive and survey-only/reactive areas respectively. However, given the demonstrated negative effects of proactive badger culling on bTB incidence in herds on land outside but within 2 km of the areas culled as well as its declining benefits inside trial areas once culling has stopped, detailed consideration is needed to determine whether (and where) proactive badger culling could be an effective part of bTB control in England and Wales. We also produce further evidence that the livestock population within 1500 m of a farm, but not counting the index herd, is associated with the risk of detecting bTB. While the randomized design of the RBCT facilitates the interpretation of treatment effects (between trial areas), its principal aim was not to assess variation in bTB risk at the herd level within trial areas. Our conclusions are therefore cautious due to the observational nature of our study.

### In conclusion

In the long-term, Defra is “considering the potential for a more risk-based approach to setting routine bTB testing intervals […] (to be) in a better position to tackle the disease” [51]. On-farm surveillance for bTB is currently carried out through a programme of routine testing, with cattle herds tested every one, two, three or four years depending on the local level of risk of infection with *M. bovis* and historic incidence (risk level reviewed annually). The measures of local bTB revealed by the present analyses could be integrated into a risk-based herd testing programme to improve the targeting of interventions aimed at reducing the risks of bTB transmission to cattle herds in areas densely populated with livestock and/or badgers.

## Supporting Information

Supporting Information S1 Univariable models and alternative multivariable models of herd-level bTB risk.
(DOC)
