# Supplementary material for: Local Cattle and Badger Populations Affect the Risk of Confirmed Tuberculosis in British Cattle Herds
Source: PLoS One. 2011 Mar 28;6(3):e18058. doi: 10.1371/journal.pone.0018058 (PMC3065452; doi:10.1371/journal.pone.0018058)

# Supplementary Information: Local cattle and badger populations affect the risk of confirmed tuberculosis in British cattle herds.

Flavie Vial^1^, W. Thomas Johnston^2^ and Christl A. Donnelly^1^

^1^ Department of Infectious Disease Epidemiology, MRC Centre for Outbreak Analysis and Modelling, School of Public Health, Imperial College London, London, United Kingdom.

^2^ Department of Health Sciences, University of York, York, United Kingdom

## The variables

### All land parcels recorded in the RBCT geodatabase (ArcGIS version 9, ESRI) were identified as being either inside or outside of the trial area boundaries and separate datasets were created for each set of parcels. Locations of all badger setts identified in the initial surveys carried out before the initial culls and all badgers culled during the proactive culls were plotted. Parcels were identified as being either herds or other occupiers (no evidence of cattle ownership). Herds were further classified by whether or not they had undergone a herd bTB test in the 12 months prior to the initial badger cull for the trial area where they were located in the case of study herds. Setts were classified as being active or inactive and main setts were identified. Badgers were classified as *M. bovis* positive or negative and by which cull (first, second, third, etc.) they were cage trapped during. The number of target herds, both inside and outside the trial area boundaries, badger setts of the various types and *M. bovis* positive and negative badgers within 500m, 1000m, 1500m of the boundaries of each study parcel was determined. These data were summarised in two ways. First, the main parcel (largest land area) for each study occupier or herd was identified and the numbers of each element determined for that parcel only. Second, the numbers of each element near to all parcels for each study occupier or herd were determined. The raw counts of herds, badgers and setts were converted to density estimates simply by dividing by the area over which these elements were counted. The variable investigated are listed in Table 1 with some basic descriptive statistics.

Table 1: The median, mean, standard error (S.E), number of observations (N), minimum (Min) and maximum (Max) of the badger, sett and herd variables inside proactive areas (A), survey-only areas (B), reactive areas (C) and within 500/1000 and 1500m^1^ of all land parcels belonging to a premise^2^.

|  | **A) Proactive** | Distance | Median | Mean | S.E | N | Min | Max |
| --- | --- | --- | --- | --- | --- | --- | --- | --- |
| **Badger**  **variables ^3^** | Total number of badgers culled | 500 | 6 | 10.15 | 0.43 | 892 | 0 | 93 |
|  |  | 1000 | 15 | 22.47 | 0.81 | 892 | 0 | 151 |
|  |  | 1500 | 26.5 | 37.58 | 1.20 | 892 | 0 | 222 |
|  | Number of badgers culled that tested positive for *M. bovis* | 500 | 0 | 1.08 | 0.07 | 892 | 0 | 17 |
|  |  | 1000 | 1 | 2.32 | 0.13 | 892 | 0 | 27 |
|  |  | 1500 | 2 | 3.83 | 0.18 | 892 | 0 | 45 |
|  | Total density of badgers culled  ( / km^2^) | 500 | 1.89 | 3.18 | 0.14 | 892 | 0 | 28.12 |
|  |  | 1000 | 2.14 | 3.23 | 0.11 | 892 | 0 | 17.64 |
|  |  | 1500 | 2.41 | 3.21 | 0.10 | 892 | 0 | 16.30 |
|  | Density badgers culled that tested positive for *M. bovis* (/ km^2^) | 500 | 0 | 0.30 | 0.02 | 892 | 0 | 4.79 |
|  |  | 1000 | 0.14 | 0.31 | 0.02 | 892 | 0 | 2.73 |
|  |  | 1500 | 0.15 | 0.31 | 0.01 | 892 | 0 | 2.21 |

|  | **A) Proactive** continues … | Distance | Median | Mean | S.E | N | Min | Max |
| --- | --- | --- | --- | --- | --- | --- | --- | --- |
| **Sett**  **variables** ^4^ | Number of active badger setts | 500 | 8 | 11.10 | 0.39 | 892 | 0 | 77 |
|  |  | 1000 | 18 | 24.03 | 0.70 | 892 | 0 | 150 |
|  |  | 1500 | 33 | 40.97 | 1.06 | 892 | 1 | 267 |
|  | Density of badger active setts (/ km^2^) | 500 | 2.29 | 2.96 | 0.10 | 892 | 0 | 19.42 |
|  |  | 1000 | 2.34 | 2.86 | 0.07 | 892 | 0 | 13.56 |
|  |  | 1500 | 2.34 | 2.81 | 0.06 | 892 | 0.1 | 11.47 |
| **Herd variables** ^5^ | Number of cattle herds tested for bTB | 500 | 7 | 7.66 | 0.15 | 892 | 0 | 52 |
|  |  | 1000 | 12 | 13.05 | 0.24 | 892 | 0 | 70 |
|  |  | 1500 | 17 | 19.48 | 0.34 | 892 | 2 | 96 |
|  | Density of cattle herds  tested for bTB  (/ km^2^) | 500 | 2 | 2.33 | 0.05 | 892 | 0 | 8.58 |
|  |  | 1000 | 1.51 | 1.68 | 0.03 | 892 | 0 | 5.52 |
|  |  | 1500 | 1.26 | 1.41 | 0.02 | 892 | 0.19 | 4.48 |

|  | **B) Survey-only** | Distance | Median | Mean | S.E | N | Min | Max |
| --- | --- | --- | --- | --- | --- | --- | --- | --- |
| **Sett**  **variables** ^4^ | Number of active badger setts | 500 | 7 | 9.64 | 0.33 | 958 | 0 | 89 |
|  |  | 1000 | 17 | 21.52 | 0.60 | 958 | 0 | 144 |
|  |  | 1500 | 30 | 36.65 | 0.91 | 958 | 2 | 198 |
|  | Density of badger active setts  (/ km^2^) | 500 | 2.05 | 2.67 | 0.08 | 958 | 0 | 19.17 |
|  |  | 1000 | 2.22 | 2.63 | 0.07 | 958 | 0 | 16.23 |
|  |  | 1500 | 2.26 | 2.56 | 0.05 | 958 | 0.17 | 13.85 |
| **Herd variables** ^5^ | Number of cattle herds tested for bTB | 500 | 7 | 7.27 | 0.13 | 958 | 0 | 27 |
|  |  | 1000 | 11 | 12.50 | 0.20 | 958 | 1 | 46 |
|  |  | 1500 | 17 | 18.39 | 0.27 | 958 | 3 | 59 |
|  | Density of cattle herds  tested for bTB (/ km^2^) | 500 | 2.00 | 2.23 | 0.04 | 958 | 0 | 9.31 |
|  |  | 1000 | 1.47 | 1.61 | 0.02 | 958 | 0.19 | 4.68 |
|  |  | 1500 | 1.20 | 1.33 | 0.02 | 958 | 0.28 | 3.38 |

|  | **C) Reactive** | Distance | Median | Mean | S.E | N | Min | Max |
| --- | --- | --- | --- | --- | --- | --- | --- | --- |
| **Sett**  **variable s** ^4^ | Number of active badger setts | 500 | 7 | 11.05 | 0.41 | 938 | 0 | 167 |
|  |  | 1000 | 17 | 23.85 | 0.74 | 938 | 0 | 295 |
|  |  | 1500 | 30 | 40.68 | 1.14 | 938 | 0 | 433 |
|  | Density of badger active setts  (/ km^2^) | 500 | 2.11 | 2.92 | 0.09 | 938 | 0 | 15.56 |
|  |  | 1000 | 2.14 | 2.89 | 0.07 | 938 | 0 | 15.25 |
|  |  | 1500 | 2.18 | 2.82 | 0.07 | 938 | 0 | 12.79 |
| **Herd variables** ^5^ | Number of cattle herds tested for bTB | 500 | 7 | 7.49 | 0.15 | 938 | 0 | 32 |
|  |  | 1000 | 12 | 12.97 | 0.22 | 938 | 0 | 49 |
|  |  | 1500 | 17 | 18.89 | 0.30 | 938 | 0 | 63 |
|  | Density of cattle herds  tested for bTB (/ km^2^) | 500 | 2.03 | 2.30 | 0.04 | 938 | 1 | 8.80 |
|  |  | 1000 | 1.53 | 1.68 | 0.03 | 938 | 0 | 4.51 |
|  |  | 1500 | 1.28 | 1.38 | 0.02 | 938 | 0.06 | 3.55 |

^1^ Numbers and densities within *x* meters reflect observations on the land parcels themselves and on the buffer of width *x* around them.

^2^ Untransformed values are presented, although these were screened after being log-transformed.

^3^ Relate to badgers culled during the initial proactive culls only.

^4^ Relate to the badger setts identified during the initial survey before herds were allocated to their treatment group.

^5^ Relate to herds tested for bTB during the one year prior to the start of the initial proactive cull.

## Local risk factors

### Herd size

## Herd size was extracted from the VetNet database (Figure 1). Herd sizes ranged from zero to 1723 head of cattle. The “zero” entries (all corresponding to herds where a bTB breakdown had occurred) were corrected using the herd size recorded by veterinary officers during the herd testing associated with the first breakdown. The median herd size was 72 head of cattle (mean = 102, standard error = 1.72).

Figure 1: Distribution of herd sizes throughout all RBCT trial areas as extracted from the VetNet database (n = 3863).


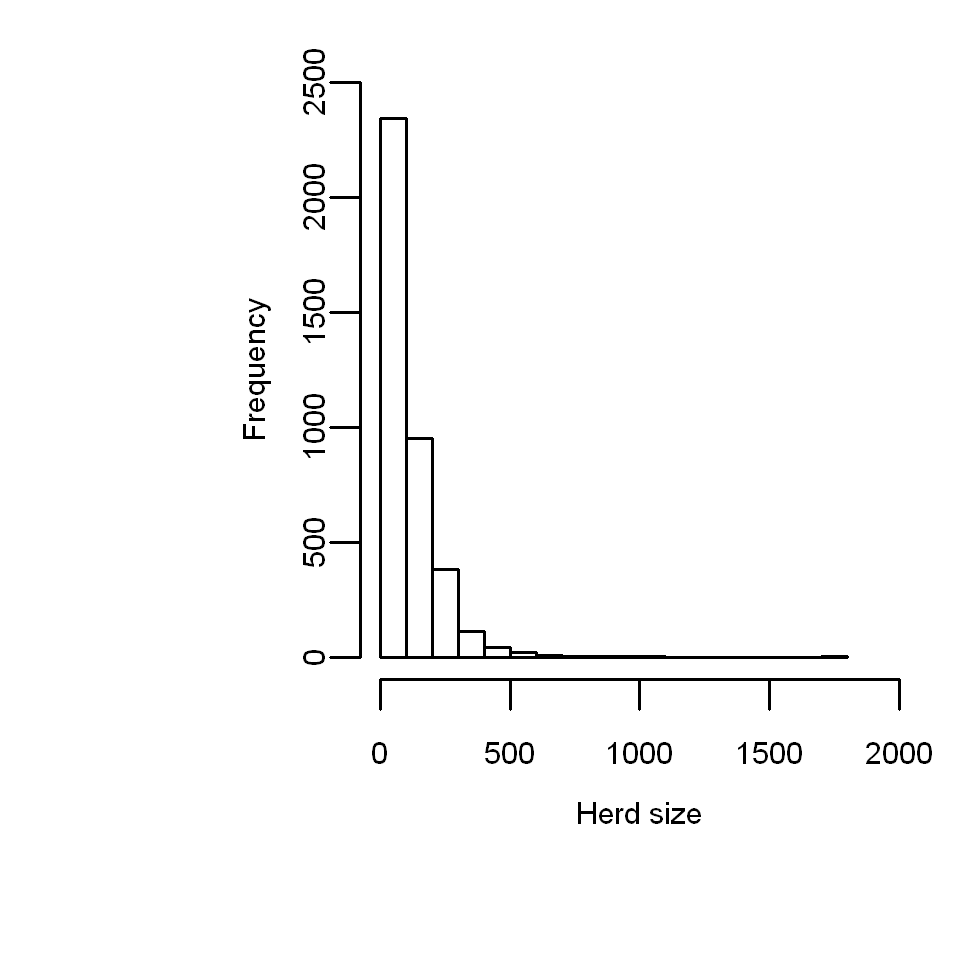


### Historic incidence and number of baseline herds

Both local-risk factors were estimated at the triplet-level. The historic incidence of cattle bTB was calculated for the three-year period before the initial proactive cull, except in triplets D, I and J where it was calculated for the three years prior to the start of the 2001 foot and mouth disease epidemic (Table 4 ). The number of baseline herds in a triplet was defined as the number of herds recorded for that triplet prior to the beginning of the initial badger cull.

Table 2: Number of baseline herds and bTB historic incidence for all triplets inside the RBCT.

|  | **Proactive area** | | **Survey-only area** | | **Reactive area** | |
| --- | --- | --- | --- | --- | --- | --- |
| **Triplet** | **Number of herds** | **Historic incidence** | **Number of herds** | **Historic incidence** | **Number of herds** | **Historic incidence** |
| **A** | 71 | 33 | 89 | 33 | 105 | 27 |
| **B** | 153 | 40 | 33 | 27 | 89 | 26 |
| **C** | 107 | 15 | 173 | 27 | 157 | 25 |
| **D** | 98 | 28 | 108 | 30 | 100 | 23 |
| **E** | 116 | 25 | 101 | 28 | 76 | 16 |
| **F** | 142 | 12 | 190 | 34 | 220 | 19 |
| **G** | 245 | 26 | 131 | 15 | 120 | 16 |
| **H** | 66 | 23 | 129 | 22 | 69 | 22 |
| **I** | 107 | 30 | 98 | 19 | 62 | 42 |
| **J** | 116 | 25 | 124 | 18 | 129 | 43 |

### Farm area and number of land parcels

Most farms operated from two land parcels (min = 1, max = 16, median = 2) and median farm area (combined area of all the land parcels belonging to a farm) was estimated at 0.50 km^2^ (min = 0.002, max = 12.38) (Figure 2).

Figure 2: Distribution of farm area (left, n = 3902) and number of land parcels per farm (right, n = 2788) throughout all RBCT trial areas .


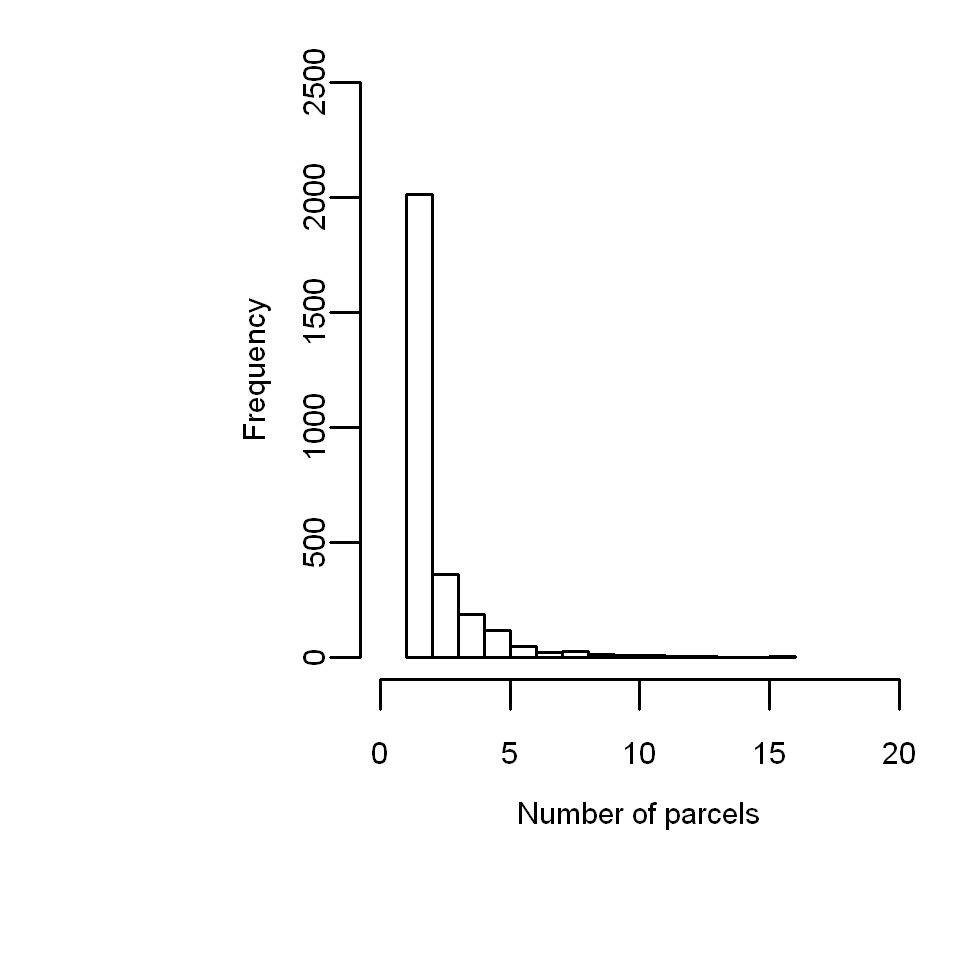

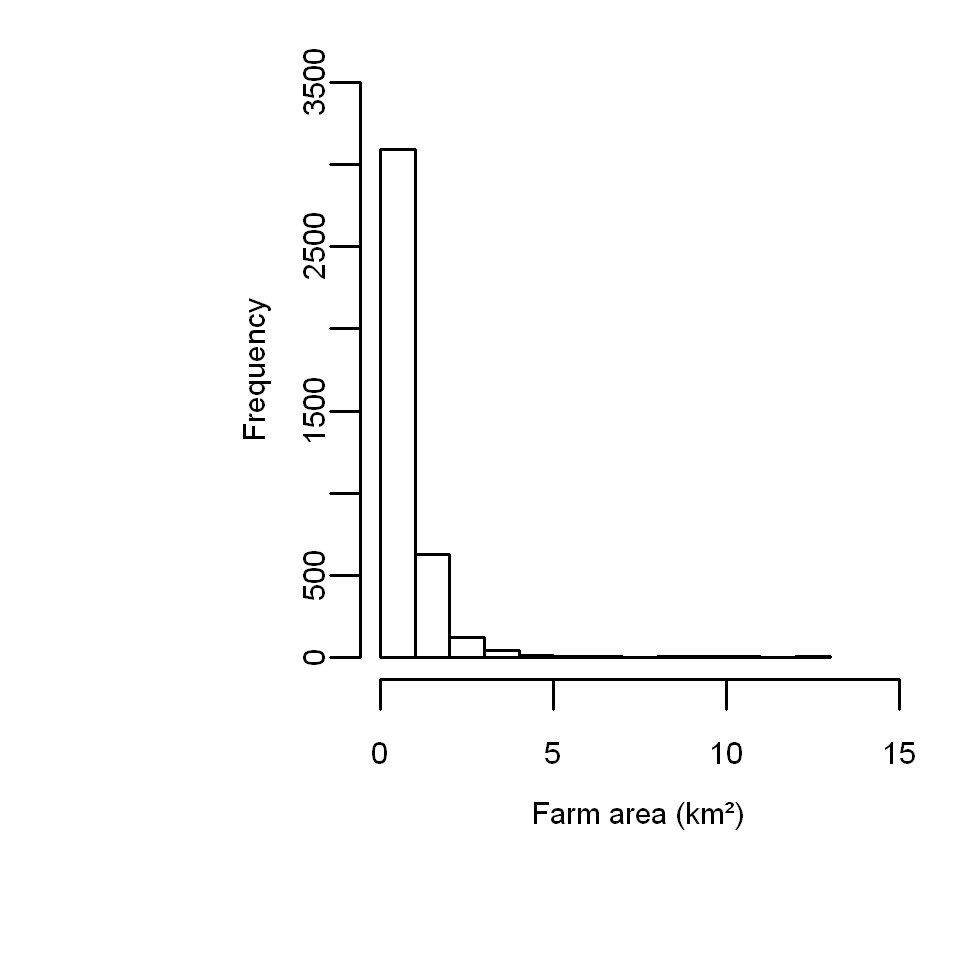


Farm area and the number of land parcels belonging to that farm were highly correlated (Pearson's product moment coefficient = 0.39, p< 0.001) (Figure 3).

Figure 3: Boxplot representing median farm area for a given number of land parcels.


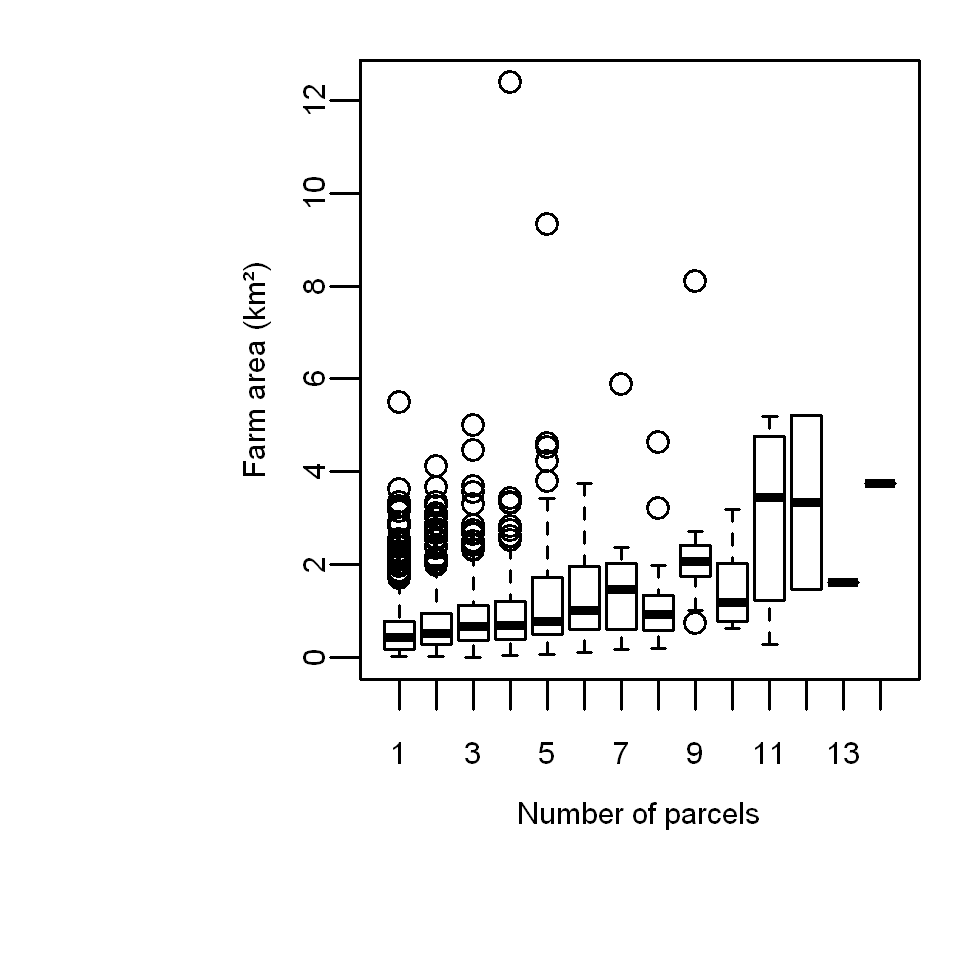


## Univariable logistic regression models

The non-categorical variables were log transformed before being individually screened using logistic regression controlling for local farm-level risks (namely herd size, herd type, farm area and bTB historic incidence within the trial area). A distinction was made for badger-related and sett-related variables between the number of badgers culled or setts recorded on the premise’s land parcels and those on the buffer surrounding the premise (Table 3). Such distinction was significant inside all treatment groups. P-values were adjusted for overdispersion, when present, by using an inflation factor equal to the square root of the model deviance divided by the degrees of freedom.

Table 3: Univariable models (adjusted for herd type, herd size, farm area and bTB historic incidence in the trial area) of the probability of RBCT herds within a proactive (A), survey-only (B) and reactive (C) trial area experiencing a confirmed bTB breakdown during the period under study.

| **A) Proactive** |  | **Outside but within *x* meters of all land parcels belonging to the farm** | | |
| --- | --- | --- | --- | --- |
|  |  | a) 500m | b) 1000m | c) 1500m |
| **Badger variables ^1^** | Number of badgers culled total | p = 0.20 | p = 0.10 | p = 0.18 |
|  |  | OR: 1.05 | OR: 1.07 | OR: 1.06 |
|  |  | (0.98-1.13) | (0.99-1.15) | (0.97-1.15) |
|  | Number of badgers culled on the land parcels | p = 0.55 | p = 0.55 | p = 0.55 |
|  |  | OR: 1.03 | OR: 1.03 | OR: 1.03 |
|  |  | (0.93-1.13) | (0.93-1.13) | (0.93-1.13) |
|  | Number of badgers culled outside but within *x* meters | p = 0.40 | p = 0.12 | p = 0.24 |
|  |  | OR 1.03 | OR: 1.07 | OR: 1.06 |
|  |  | (0.96-1.11) | (0.98-1.15) | (0.97-1.15) |
|  | Number of *M. bovis* + badgers culled total | **p<0.001** | **p<0.001** | **p<0.001** |
|  |  | OR: 1.24 | OR: 1.23 | OR: 1.18 |
|  |  | (1.12-1.35) | (1.13-1.33) | (1.09-1.27) |
|  | Number of *M. bovis* + badgers culled on the land | p = 0.17 | p = 0.17 | p = 0.17 |
|  | parcels | OR: 1.16 | OR: 1.16 | OR: 1.16 |
|  |  | (0.95-1.37) | (0.95-1.37) | (0.95-1.37) |

| **A) Proactive** | continues… | **Outside but within *x* meters of all land parcels belonging to the farm** | | |
| --- | --- | --- | --- | --- |
| **Badger variables ^1^** | Number of *M. bovis* + badgers culled outside | p = 0.002 | p<0.001 | p = 0.002 |
|  | but within *x* meters | OR: 1.22 | OR: 1.21 | OR: 1.16 |
|  |  | (1.10-1.35) | (1.11-1.31) | (1.07-1.25) |
|  | Density (/ km^2^) of badgers culled total | p = 0.82 | p = 0.71 | p = 0.93 |
|  |  | OR: 0.99 | OR: 1.02 | OR: 1.00 |
|  |  | (0.91-1.07) | (0.93-1.10) | (0.91-1.10) |
|  | Density (/km^2^) of badgers culled on the land parcels | p = 0.06 | p = 0.06 | p = 0.06 |
|  |  | OR: 0.91 | OR: 0.91 | OR: 0.91 |
|  |  | (0.81-1.01) | (0.81-1.01) | (0.81-1.01) |
|  | Density (/km^2^) of badgers culled outside | p = 0.54 | p = 0.78 | p = 0.98 |
|  | but within *x* meters | OR: 0.98 | OR: 1.01 | OR: 1.00 |
|  |  | (0.90-1.06) | (0.93-1.10) | (0.90-1.09) |
|  |  | a) 500m | b) 1000m | c) 1500m |
|  | Density (/km^2^) of *M. bovis* + badgers culled total | p = 0.17 | **p = 0.006** | **p = 0.02** |
|  |  | OR: 1.09 | OR: 1.15 | OR: 1.13 |
|  |  | (0.97-1.20) | (1.05-1.25) | (1.03-1.22) |
|  | Density (/km^2^) of *M. bovis* + badgers culled | **p = 0.003** | **p = 0.003** | **p = 0.003** |
|  | on the land parcels | OR: 0.76 | OR: 0.76 | OR: 0.76 |
|  |  | (0.59-0.94) | (0.59-0.94) | (0.59-0.94) |
|  | Density (/km^2^) of *M. bovis* + badgers culled | p = 0.42 | **p = 0.03** | p = 0.05 |
|  | outside but within *x* meters | OR: 1.13 | OR: 1.13 | OR: 0.91 |
|  |  | (1.00-1.26) | (1.02-1.23) | (0.81-1.00) |
| **Sett variables ^2^** | Number of active setts total | p = 0.03 | p = 0.005 | p = 0.03 |
|  |  | OR: 1.14 | OR: 1.21 | OR: 1.18 |
|  |  | (1.02-1.25) | (1.07-1.34) | (1.04-1.33) |
|  | Number of active setts on the land parcels | p = 0.07 | p = 0.07 | p = 0.07 |
|  |  | OR: 1.12 | OR: 1.12 | OR: 1.12 |
|  |  | (1.00-1.24) | (1.00-1.24) | (1.00-1.24) |
|  | Number of active setts outside but within *x* meters | p = 0.06 | p = 0.01 | p = 0.03 |
|  |  | OR: 1.12 | OR: 1.20 | OR: 1.18 |
|  |  | (1.00-1.24) | (1.06-1.33) | (1.03-1.34) |
|  | Density (/km^2^) of active setts total | p = 0.68 | p = 0.17 | p = 0.36 |
|  |  | OR: 1.03 | OR: 1.10 | OR: 1.08 |
|  |  | (0.91-1.14) | (0.96-1.24) | (0.92-1.23) |
|  | Density (/km^2^) of active setts on the land parcels | p = 0.19 | p = 0.19 | p = 0.19 |
|  |  | OR: 0.92 | OR: 0.92 | OR: 0.92 |
|  |  | (0.80-1.04) | (0.80-1.04) | (0.80-1.04) |
|  | Density (/km^2^) of active setts outside but | p = 0.65 | p = 0.18 | p = 0.32 |
|  | within *x* meters | OR: 1.03 | OR: 1.10 | OR: 1.09 |
|  |  | (0.91-1.15) | (0.96-1.25) | (0.92-1.25) |
| **Herd variables ^3^** | Number of cattle herds tested | p = 0.25 | p = 0.43 | p = 0.53 |
|  |  | OR: 1.13 | OR: 1.09 | OR: 1.07 |
|  |  | (0.92-1.33) | (0.88-1.30) | (0.85-1.29) |
|  | Density (/km^2^) of cattle herds  Tested | **p = 0.02** | **p = 0.048** | p = 0.10 |
|  |  | OR: 0.77 | OR: 0.79 | OR: 0.81 |
|  |  | (0.54-0.99) | (0.55-1.00) | (0.57-1.06) |

| **B) Survey-only** |  | **Outside but within *x* meters of all land parcels belonging to the farm** | | |
| --- | --- | --- | --- | --- |
| **Sett variables ^2^** | Number of active setts total | **p = 0.001** | **p = 0.001** | **p = 0.001** |
|  |  | OR: 1.19 | OR: 1.21 | OR: 1.24 |
|  |  | (1.08-1.29) | (1.10-1.33) | (1.11-1.36) |
|  | Number of active setts on the land parcels | p = 0.89 | p = 0.89 | p = 0.89 |
|  |  | OR: 1.01 | OR: 1.01 | OR: 1.01 |
|  |  | (0.89-1.12) | (0.89-1.12) | (0.89-1.12) |
|  | Number of active setts outside but within *x* meters | **p = 0.002** | **p = 0.002** | **p = 0.002** |
|  |  | OR: 1.18 | OR: 1.21 | OR: 1.23 |
|  |  | (1.07-1.28) | (1.09-1.33) | (1.10-1.36) |
|  | Density (/km^2^) of active setts total | p = 0.09 | **p = 0.04** | **p = 0.03** |
|  |  | OR: 1.10 | OR: 1.13 | OR: 1.16 |
|  |  | (0.99-1.20) | (1.02-1.26) | (1.03-1.29) |
|  | Density (/km^2^) of active setts on the land parcels | **p = 0.02** | **p = 0.02** | **p = 0.02** |
|  |  | OR: 0.87 | OR: 0.87 | OR: 0.87 |
|  |  | (0.75-0.98) | (0.75-0.98) | (0.75-0.98) |
|  | Density (/km^2^) of active setts outside but | p = 0.10 | **p = 0.05** | **p = 0.04** |
|  | within *x* meters | OR: 1.09 | OR: 1.13 | OR: 1.15 |
|  |  | (0.99-1.20) | (1.01-1.25) | (1.02-1.29) |
| **Herd variables ^3^** | Number of cattle herds tested | **p = 0.001** | **p<0.001** | **p = 0.004** |
|  |  | OR: 1.41 | OR: 1.46 | OR: 1.42 |
|  |  | (1.21-1.62) | (1.24-1.68) | (1.18-1.65) |
|  | Density (/km^2^) of cattle herds  Tested | p = 0.87 | p = 0.30 | p = 0.42 |
|  |  | OR: 1.02 | OR: 1.14 | OR: 1.11 |
|  |  | (0.80-1.24) | (0.90-1.37) | (0.85-1.37) |

| **C) Reactive** |  | **Outside but within *x* meters of all land parcels belonging to the farm** | | |
| --- | --- | --- | --- | --- |
| **Sett variables ^2^** | Number of active setts total | p = 0.61 | p = 0.94 | p = 0.88 |
|  |  | OR: 1.03 | OR: 1.00 | OR: 10.99 |
|  |  | (0.92-1.13) | (0.88-1.13) | (0.85-1.12) |
|  | Number of active setts on the land parcels | p = 0.44 | p = 0.44 | p = 0.44 |
|  |  | OR: 1.04 | OR: 1.04 | OR: 1.04 |
|  |  | (0.93-1.15) | (0.93-1.15) | (0.93-1.15) |
|  | Number of active setts outside but within *x* meters | p = 0.53 | p = 0.93 | p = 0.98 |
|  |  | OR: 1.04 | OR: 1.01 | OR: 1.00 |
|  |  | (0.93-1.14) | (0.88-1.13) | (0.86-1.14) |
|  | Density (/km^2^) of active setts total | p = 0.15 | p = 0.13 | p = 0.15 |
|  |  | OR: 0.99 | OR: 0.91 | OR: 0.90 |
|  |  | (0.88 -1.10) | (0.78-1.03) | (0.76-1.04) |
|  | Density (/km^2^) of active setts on the land parcels | **p = 0.03** | **p = 0.03** | **p = 0.03** |
|  |  | OR: 0.88 | OR: 0.88 | OR: 0.88 |
|  |  | (0.77-0.99) | (0.77-0.99) | (0.77-0.99) |
|  | Density (/km^2^) of active setts outside but | p = 0.26 | p = 0.16 | p = 0.21 |
|  | within *x* meters | OR: 0.94 | OR: 0.91 | OR: 0.91 |
|  |  | (0.83-1.05) | (0.78-1.04) | (0.77-1.06) |

| **C) Reactive** | continues… | **Outside but within *x* meters of all land parcels belonging to the farm** | | |
| --- | --- | --- | --- | --- |
| **Herd variables ^3^** | Number of cattle herds tested | **p<0.001** | **p<0.001** | **p<0.001** |
|  |  | OR: 1.72 | OR: 1.78 | OR: 1.74 |
|  |  | (1.53-1.92) | (1.57-1.99) | (1.52-1.95) |
|  | Density (/km^2^) of cattle herds  Tested | **p = 0.005** | **p<0.001** | **p<0.001** |
|  |  | OR: 1.34 | OR: 1.49 | OR: 1.53 |
|  |  | (1.14-1.54) | (1.26-1.72) | (1.29-1.76) |

A distinction is made for herds for which consent to survey and/or cull was granted between badger-related and sett-related variables on the land parcels belonging to the farm and the buffer area surrounding them.

Odds ratios (OR) are quoted with their corresponding 95% confidence interval, and for covariates correspond to the change in the risk of a confirmed bTB breakdown following a doubling of the value of the covariate.

^1^ Relate to the badgers culled during the initial proactive cull.

^2^ Relate to the badger setts identified during the initial survey before herds were allocated to their treatment group.

^3^ Relate to herds tested for bTB during the one year prior to the start of the initial proactive cull.

## Multivariable logistic regression models

The badger-related, sett-related and herd-related variables which demonstrated the most significant univariable associations with the risk of confirmed herd breakdowns were retained for multivariable model building (Tables 4 & 5). P-values were adjusted for overdispersion, when present, by using an inflation factor equal to the square root of the model deviance divided by the degrees of freedom. Models were constructed by backward elimination, starting with a full model with quadratic terms for each non-categorical variable. Variables were eliminated on the basis of their significance in the model as well as their contribution to the variation in the data by means of an analysis of variance using an F test.

Table 4: Multivariable models, using predictors within 1000m of all land parcels, of the probability of RBCT herds experiencing a confirmed bTB breakdown during the period under study.

|  | **Survey-only** | | **Proactive** | | **Reactive** | |
| --- | --- | --- | --- | --- | --- | --- |
|  | Model A ^1^ | Model B | Model A | Model B | Model A | Model B |
| Number of *M. bovis* + badgers culled on the land parcels ^2^ | NA | NA | ---- | ---- | NA | NA |
| Number of *M. bovis* + badgers culled outside but within 1000m | NA | NA | p<0.001 | p = 0.002 | NA | NA |
|  |  |  | OR: 1.25 | OR: 1.21 |  |  |
|  |  |  | (1.15-1.35) | (1.11-1.31) |  |  |
| Number of active setts on the land parcels ^3^ | ---- | ---- | ---- | ---- | NA | NA |
| Number of active setts outside but within 1000m | ---- | p = 0.03 | ---- | ---- | NA | NA |
|  |  | OR: 1.15 |  |  |  |  |
|  |  | (1.03 - 1.27) |  |  |  |  |
| Density (/km^2^) of active setts on the land parcels | NA | NA | NA | NA | ---- | ---- |
| Density (/km^2^) of active setts outside but within 1000m | NA | NA | NA | NA | ---- | ---- |
| Number of cattle herds tested ^4^ | p<0.001 | p = 0.005 | NA | NA | p < 0.001 | p < 0.001 |
|  | OR: 1.60 | OR: 1.41 |  |  | OR: 1.72 | OR: 1.81 |
|  | (1.38 -1.83) | (1.17 -1.65) |  |  | (1.50 -1.93) | (1.59 -2.03) |
| Density (/km^2^) of cattle herds tested | NA | NA | ---- | ---- | NA | NA |
| Herd type [DAIRY] | p = 0.48 | p = 0.23 | p = 0.03 | p = 0.04 | p = 0.12 | p = 0.14 |
|  | OR: 1.14 | OR: 1.23 | OR: 1.46 | OR: 1.43 | OR: 0.76 | OR: 0.76 |
|  | (0.82 -1.59) | (0.88-1.72) | (1.04-2.04) | (1.02-2.02) | (0.53 -1.07) | (0.53-1.09) |
| Herd size | p < 0.001 | p < 0.001 | p = 0.002 | p = 0.001 | p < 0.001 | p < 0.001 |
|  | OR: 1.20 | OR: 1.20 | OR: 1.18 | OR: 1.19 | OR: 1.72 | OR: 1.25 |
|  | (1.10-1.31) | (1.10-1.31) | (1.07-1.29) | (1.09-1.30) | (1.50-1.93) | (1.14-1.36) |
| Farm area | p<0.001 | p < 0.001 | p <0.001 | p <0.001 | p = 0.001 | p = 0.003 |
|  | OR: 1.28 | OR: 1.29 | OR: 1.30 | OR: 1.33 | OR: 1.23 | OR: 1.21 |
|  | (1.16-1.41) | (1.16-1.41) | (1.17-1.43) | (1.20-1.46) | (1.10-1.36) | (1.08-1.34) |
| bTB historic incidence within trial area | NA | p <0.001 | NA | p <0.001 | NA | p = 0.003 |
|  |  | OR: 2.11 |  | OR: 2.46 |  | OR: 1.67 |
|  |  | (1.69-2.54) |  | (2.09-2.83) |  | (1.33-2.01) |

Odds ratios (OR) are quoted with their corresponding 95% confidence interval, and for covariates correspond to the change in the risk of a confirmed bTB breakdown following a doubling of the value of the covariate.

The --- means that an individual predictor was not significant and removed from the model, while NA corresponds to variables that were not included following the screening process.

^1^ Models are adjusted for herd size, herd type and farm area (model A); herd size, herd type, farm area and bTB historic incidence within the trial area (model B).

^2^ Relate to the badgers culled during the initial proactive cull.

^3^ Relate to the badger setts identified during the initial survey.

^4^ Relate to herds tested for bTB during the one year prior to the start of the initial proactive cull.

Table 5: Multivariable models, using predictors within 1500m of all land parcels, of the probability of RBCT herds experiencing a confirmed bTB breakdown during the period under study.

|  | **Survey-only** | | **Proactive** | | **Reactive** | |
| --- | --- | --- | --- | --- | --- | --- |
|  | Model A ^1^ | Model B | Model A | Model B | Model A | Model B |
| Number of *M. bovis* + badgers culled on the land parcels ^2^ | NA | NA | ---- | ---- | NA | NA |
| Number of *M. bovis* + badgers culled outside but within 1500m | NA | NA | p<0.001 | p = 0.002 | NA | NA |
|  |  |  | OR: 1.20 | OR: 1.16 |  |  |
|  |  |  | (1.11-1.30) | (1.07-1.25) |  |  |
| Number of active setts on the land parcels ^3^ | ---- | ---- | ---- | ---- | NA | NA |
| Number of active setts outside but within 1500m | p = 0.05 | p = 0.02 | ---- | ---- | NA | NA |
|  | OR: 1.14 | OR: 1.17 |  |  |  |  |
|  | (1.01 - 1.28) | (1.03 - 1.31) |  |  |  |  |
| Density (/km^2^) of active setts on the land parcels | NA | NA | NA | NA | ---- | ---- |
| Density (/km^2^) of active setts outside but within 1500m | NA | NA | NA | NA | ---- | ---- |
| Number of cattle herds tested ^4^ | p = 0.002 | p = 0.02 | NA | NA | p < 0.001 | p < 0.001 |
|  | OR: 1.50 | OR: 1.37 |  |  | OR: 1.67 | OR: 1.77 |
|  | (1.25 -1.75) | (1.12 -1.63) |  |  | (1.45 -1.89) | (1.54 -2.00) |
| Density (/km^2^) of cattle herds tested | NA | NA | ---- | ---- | NA | NA |
| Herd type [DAIRY] | p = 0.44 | p = 0.24 | p = 0.04 | p = 0.05 | p = 0.12 | p = 0.14 |
|  | OR: 1.14 | OR: 1.23 | OR: 1.43 | OR: 1.41 | OR: 0.76 | OR: 0.76 |
|  | (0.80 -1.54) | (0.87-1.72) | (1.02-2.00) | (1.00-1.99) | (0.53 -1.07) | (0.53-1.09) |
| Herd size | p < 0.001 | p < 0.001 | p = 0.001 | p< 0.001 | p < 0.001 | p < 0.001 |
|  | OR: 1.20 | OR: 1.21 | OR: 1.19 | OR: 1.20 | OR: 1.25 | OR: 1.26 |
|  | (1.10-1.30) | (1.10-1.31) | (1.08-2.30) | (1.09-1.31) | (1.14-1.36) | (1.15-1.36) |
| Farm area | p<0.001 | p < 0.001 | p <0.001 | p <0.001 | p<0.001 | p = 0.001 |
|  | OR: 1.27 | OR: 1.30 | OR: 1.31 | OR: 1.34 | OR: 1.26 | OR: 1.24 |
|  | (1.15-1.40) | (1.18-1.43) | (1.18-1.44) | (1.21-1.47) | (1.13-1.38) | (1.11-1.37) |
| bTB historic incidence within trial area | NA | p <0.001 | NA | p <0.001 | NA | p = 0.003 |
|  |  | OR: 2.10 |  | OR: 2.46 |  | OR: 1.67 |
|  |  | (1.68-2.52) |  | (2.09-2.84) |  | (1.33-2.01) |

Odds ratios (OR) are quoted with their corresponding 95% confidence interval, and for covariates correspond to the change in the risk of a confirmed bTB breakdown following a doubling of the value of the covariate.

The --- means that an individual predictor was not significant and removed from the model, while NA corresponds to variables that were not included following the screening process.

^1^ Models are adjusted for herd size, herd type and farm area (model A); herd size, herd type, farm area and bTB historic incidence within the trial area (model B).

^2^ Relate to the badgers culled during the initial proactive cull.

^3^ Relate to the badger setts identified during the initial survey.

^4^ Relate to herds tested for bTB during the one year prior to the start of the initial proactive cull.

The effects of triplet and historic bTB incidence within the trial areas could be not be simultaneously estimated inside each treatment group as a lack of statistical power, and separate multivariable models were tested adjusting for herd type, herd size, farm area and triplet (Table 6).

Table 6: Alternative multivariable models (adjusted for herd size, herd type, farm area and triplet) of the probability of RBCT herds experiencing a confirmed bTB breakdown during the period under study within a survey-only, proactive or reactive area.

|  | **Survey-only** | | | **Proactive** | | | **Reactive** | | |
| --- | --- | --- | --- | --- | --- | --- | --- | --- | --- |
|  | 500m | 1000m | 1500m | 500m | 1000m | 1500m | 500m | 1000m | 1500m |
| Number of *M. bovis* + badgers culled on land parcels ^1^ | NA | NA | NA | ---- | ---- |  | NA | NA | NA |
| Number of *M. bovis* + badgers culled | NA | NA | NA | p = 0.003 | p<0.001 | p<0.001 | NA | NA | NA |
| outside but within *x* m |  |  |  | OR: 1.24 | OR: 1.29 | OR: 1.26 |  |  |  |
|  |  |  |  | (1.10-1.39) | (1.16-1.41) | (1.13-1.39) |  |  |  |
| Number of active setts on the land parcels ^2^ | ---- | ---- | ---- | ---- | ---- | ---- | NA | NA | NA |
| Number of active setts outside but within *x* m | p = 0.05 | ---- | ---- | ---- | ---- | ---- | NA | NA | NA |
|  | OR: 1.14 |  |  |  |  |  |  |  |  |
|  | (1.01 - 1.28) |  |  |  |  |  |  |  |  |
| Density (/km^2^) of active setts on the land parcels | NA | NA | NA | NA | NA | NA | ---- | ---- | ---- |
| Density (/km^2^) of active setts outside but within *x* m | NA | NA | NA | NA | NA | NA | ---- | ---- | ---- |
| Number of cattle herds tested ^3^ | p<0.001 | p<0.001 | p<0.001 | NA | NA | NA | p < 0.001 | p < 0.001 | p < 0.001 |
|  | OR: 1.53 | OR: 1.82 | OR: 1.92 |  |  |  | OR: 1.91 | OR: 2.16 | OR: 2.19 |
|  | (1.28 -1.77) | (1.55 -2.09) | (1.6-2.23) |  |  |  | (1.67 -2.15) | (1.88 -2.44) | (1.89 -2.49) |
| Density (/km^2^) of cattle herds tested | NA | NA | NA | ---- | ---- | ---- | NA | NA | NA |
| Herd type [DAIRY] | p = 0.28 | p = 0.28 | p = 0.30 | p = 0.006 | p = 0.004 | p = 0.005 | p = 0.49 | p = 0.35 | p = 0.42 |
|  | OR: 1.21 | OR: 1.21 | OR: 1.20 | OR: 1.66 | OR: 1.72 | OR: 1.69 | OR: 0.87 | OR: 0.86 | OR: 0.86 |
|  | (0.85 -1.71) | (0.85 -1.71) | (0.85 -1.7) | (1.16-2.39) | (1.19-2.48) | (1.17-2.43) | (0.60 -1.28) | (0.59-1.26) | (0.58-1.25) |
| Herd size | p < 0.001 | p = 0.002 | p = 0.002 | p = 0.002 | p = 0.003 | p = 0.002 | p < 0.001 | p < 0.001 | p < 0.001 |
|  | OR: 1.20 | OR: 1.19 | OR: 1.19 | OR: 1.19 | OR: 1.19 | OR: 1.20 | OR: 1.27 | OR: 1.26 | OR: 1.26 |
|  | (1.09-1.31) | (1.08-1.30) | (1.09-1.3) | (1.08-2.30) | (1.08-1.30) | (1.09-1.31) | (1.16-1.38) | (1.15-1.38) | (1.14-1.37) |
| Farm area | p = 0.004 | p < 0.001 | p < 0.001 | p <0.001 | p <0.001 | p <0.001 | p = 0.12 | p = 0.006 | p = 0.004 |
|  | OR: 1.23 | OR: 1.29 | OR: 1.29 | OR: 1.29 | OR: 1.29 | OR: 1.30 | OR: 1.12 | OR: 1.11 | OR: 1.17 |
|  | (1.09-1.37) | (1.15-1.42) | (1.15-1.4) | (1.15-1.43) | (1.15-1.43) | (1.16-1.44) | (0.98-1.27) | (1.00-1.29) | (1.03-1.32) |
| triplet | p <0.001 | p <0.001 | p <0.001 | p <0.001 | p <0.001 | p <0.001 | p <0.001 | p <0.001 | p <0.001 |

Odds ratios (OR) are quoted with their corresponding 95% confidence interval, and for covariates correspond to the change in the risk of a confirmed bTB breakdown following a doubling of the value of the covariate.

The --- means that an individual predictor was not significant and removed from the model, while NA corresponds to variables that were not included following the screening process.

^1^ Relate to the badgers culled during the initial proactive cull.

^2^Relate to the badger setts identified during the initial survey.

^3^ Relate to herds tested for bTB during the one year prior to the start of the initial proactive cull.

1. **Univariable PH models**

Transformed variables were individually screened using PH models controlling for local farm-level risks (namely herd size, herd type, farm area and historic incidence). A distinction was once again made for badger-related and sett-related variables between the number of badgers culled or setts recorded on the premise’s land parcels and those on the buffer surrounding the premise (Table 7). Such distinction was only significant inside proactive and survey-only trial areas and will only be retained in the multivariable models for these herds (not for herds inside reactive areas).

Table 7: Univariable models (adjusted for herd type, herd size, farm area and bTB historic incidence in the trial area) of the time to first confirmed bTB breakdown for RBCT herds within a proactive (A), survey-only (B) and reactive (C) trial area during the period under study.

| **A) Proactive** |  | **Outside but within *x* meters of all land parcels belonging to the farm** | | |
| --- | --- | --- | --- | --- |
|  |  | a) 500m | b) 1000m | c) 1500m |
| **Badger variables ^1^** | Number of badgers culled total | **p = 0.04** | **p = 0.02** | p = 0.21 |
|  |  | HR: 1.06 | HR: 1.07 | HR: 1.05 |
|  |  | (1.00-1.12) | (1.02-1.13) | (0.98-1.11) |
|  | Number of badgers culled on the land parcels | p = 0.21 | p = 0.21 | p = 0.21 |
|  |  | HR: 1.05 | HR: 1.05 | HR: 1.05 |
|  |  | (0.98-1.11) | (0.98-1.11) | (0.98-1.11) |
|  | Number of badgers culled outside but within *x* meters | p = 0.08 | **p = 0.02** | **p = 0.03** |
|  |  | HR 1.05 | HR: 1.07 | HR: 1.07 |
|  |  | (1.00-1.11) | (1.01-1.13) | (1.01-1.14) |
|  | Number of *M. bovis* + badgers culled total | **p<0.001** | **p<0.001** | **p<0.001** |
|  |  | HR: 1.26 | HR: 1.26 | HR: 1.23 |
|  |  | (1.18-1.34) | (1.19-1.33) | (1.08-1.36) |
|  | Number of *M. bovis* + badgers culled on the land | **p = 0.006** | **p = 0.006** | **p = 0.006** |
|  | parcels | HR: 1.23 | HR: 1.23 | HR: 1.23 |
|  |  | (1.08-1.36) | (1.08-1.36) | (1.08-1.36) |
|  | Number of *M. bovis* + badgers culled outside | **p<0.001** | **p<0.001** | **p<0.001** |
|  | but within *x* meters | HR: 1.25 | HR: 1.24 | HR: 1.21 |
|  |  | (1.17-1.34) | (1.17-1.32) | (1.14-1.28) |
|  | Density (/ km^2^) of badgers culled total | p = 0.55 | p = 0.22 | p = 0.30 |
|  |  | HR: 1.02 | HR: 1.04 | HR: 1.04 |
|  |  | (0.96-1.07) | (0.97-1.10) | (0.97-1.10) |
|  | Density (/km^2^) of badgers culled on the land parcels | p = 0.15 | p = 0.15 | p = 0.15 |
|  |  | HR: 0.59 | HR: 0.59 | HR: 0.59 |
|  |  | (0.52-0.66) | (0.52-0.66) | (0.52-0.66) |
|  | Density (/km^2^) of badgers culled outside | p = 0.78 | p = 0.24 | p = 0.32 |
|  | but within *x* meters | HR: 1.01 | HR: 1.04 | HR: 1.04 |
|  |  | (0.95-1.07) | (0.97-1.10) | (0.97-1.10) |
|  | Density (/km^2^) of *M. bovis* + badgers culled total | **p < 0.001** | **p<0.001** | **p<0.001** |
|  |  | HR: 1.16 | HR: 1.22 | HR: 1.20 |
|  |  | (1.07-1.25) | (1.14-1.30) | (1.12-1.27) |
|  | Density (/km^2^) of *M. bovis* + badgers culled | **p = 0.02** | **p = 0.02** | **p = 0.02** |
|  | on the land parcels | HR: 0.85 | HR: 0.85 | HR: 0.85 |
|  |  | (0.71-0.98) | (0.71-0.98) | (0.71-0.98) |
|  | Density (/km^2^) of *M. bovis* + badgers culled | **p = 0.01** | **p<0.001** | **p<0.001** |
|  | outside but within *x* meters | HR: 1.13 | HR: 1.20 | HR: 1.18 |
|  |  | (1.04-1.22) | (1.12-1.28) | (1.10-1.26) |

| **A) Proactive** | continues… | **Outside but within *x* meters of all land parcels belonging to the farm** | | |
| --- | --- | --- | --- | --- |
| **Sett variables ^2^** | Number of active setts total | **p<0.001** | **p <0.001** | **p = 0.003** |
|  |  | HR: 1.13 | HR: 1.18 | HR: 1.18 |
|  |  | (1.04-1.22) | (1.09-1.28) | (1.07-1.29) |
|  | Number of active setts on the land parcels | **p = 0.01** | **p = 0.01** | **p = 0.01** |
|  |  | HR: 1.11 | HR: 1.11 | HR: 1.11 |
|  |  | (1.03-1.20) | (1.03-1.20) | (1.03-1.20) |
|  | Number of active setts outside but within *x* meters | **p = 0.02** | **p = 0.001** | **p = 0.003** |
|  |  | HR: 1.11 | HR: 1.18 | HR: 1.18 |
|  |  | (1.03-1.19) | (1.08-1.27) | (1.07-1.28) |
|  | Density (/km^2^) of active setts total | p = 0.29 | **p = 0.03** | p = 0.06 |
|  |  | HR: 1.05 | HR: 1.12 | HR: 1.11 |
|  |  | (0.96-1.14) | (1.02-1.22) | (1.00-1.23) |
|  | Density (/km^2^) of active setts on the land parcels | p = 0.44 | p = 0.44 | p = 0.44 |
|  |  | HR: 0.97 | HR: 0.97 | HR: 0.97 |
|  |  | (0.88-1.05) | (0.88-1.05) | (0.88-1.05) |
|  | Density (/km^2^) of active setts outside but | p = 0.30 | **p = 0.03** | p = 0.05 |
|  | within *x* meters | HR: 1.05 | HR: 1.12 | HR: 1.12 |
|  |  | (0.96-1.14) | (1.02-1.23) | (1.01-1.24) |
| **Herd variables ^3^** | Number of cattle herds tested | p = 0.75 | p = 0.91 | p = 0.73 |
|  |  | HR: 1.02 | HR: 0.99 | HR: 10.97 |
|  |  | (0.88-1.17) | (0.84-1.14) | (0.81-1.13) |
|  | Density (/km^2^) of cattle herds  Tested | **p<0.001** | **p = 0.001** | **p = 0.003** |
|  |  | HR: 0.74 | HR: 0.75 | HR: 0.76 |
|  |  | (0.58-0.91) | (0.57-0.92) | (0.58-0.94) |

| **B) Survey-only** |  | **Outside but within *x* meters of all land parcels belonging to the farm** | | |
| --- | --- | --- | --- | --- |
| **Sett variables ^2^** | Number of active setts total | **p = 0.006** | **p = 0.005** | **p = 0.005** |
|  |  | HR: 1.11 | HR: 1.13 | HR: 1.15 |
|  |  | (1.04-1.19) | (1.05-1.22) | (1.06-1.25) |
|  | Number of active setts on the land parcels | p = 0.85 | p = 0.85 | p = 0.85 |
|  |  | HR: 1.01 | HR: 1.01 | HR: 1.01 |
|  |  | (0.93-1.09) | (0.93-1.09) | (0.93-1.09) |
|  | Number of active setts outside but within *x* meters | **p = 0.006** | **p = 0.004** | **p = 0.003** |
|  |  | HR: 1.12 | HR: 1.14 | HR: 1.16 |
|  |  | (1.04-1.20) | (1.05-1.23) | (1.06-1.26) |
|  | Density (/km^2^) of active setts total | p = 0.25 | p = 0.11 | p = 0.08 |
|  |  | HR: 1.05 | HR: 1.08 | HR: 1.09 |
|  |  | (0.97-1.13) | (0.99-1.17) | (0.99-1.09) |
|  | Density (/km^2^) of active setts on the land parcels | **p = 0.003** | **p = 0.003** | **p = 0.02** |
|  |  | HR: 1.26 | HR: 1.27 | HR: 1.23 |
|  |  | (1.11-1.41) | (1.11-1.43) | (1.06-1.40) |
|  | Density (/km^2^) of active setts outside but | p = 0.19 | p = 0.10 | p = 0.06 |
|  | within *x* meters | HR: 1.06 | HR: 1.08 | HR: 1.10 |
|  |  | (0.98-1.14) | (0.99-1.17) | (1.00-1.20) |

| **B) Survey-only** | continues… | **Outside but within *x* meters of all land parcels belonging to the farm** | | |
| --- | --- | --- | --- | --- |
| **Herd variables ^3^** | Number of cattle herds tested | **p = 0.003** | **p = 0.003** | **p = 0.02** |
|  |  | HR: 1.261 | HR: 1.27 | HR: 1.23 |
|  |  | (1.11-1.41) | (1.11-1.43) | (1.06-1.40) |
|  | Density (/km^2^) of cattle herds  Tested | p = 0.77 | p = 0.62 | p = 0.89 |
|  |  | HR: 0.98 | HR: 1.05 | HR: 1.01 |
|  |  | (0.81-1.14) | (0.87-1.22) | (0.82-1.20) |

| **C) Reactive** |  | **Outside but within *x* meters of all land parcels belonging to the farm** | | |
| --- | --- | --- | --- | --- |
| **Sett variables ^2^** | Number of active setts total | **p = 0.004** | p = 0.08 | p = 0.08 |
|  |  | HR: 1.09 | HR: 1.09 | HR: 1.09 |
|  |  | (1.01-1.17) | (0.99-1.18) | (0.99-1.19) |
|  | Number of active setts on the land parcels | p = 0.09 | p = 0.09 | p = 0.09 |
|  |  | HR: 1.07 | HR: 1.07 | HR: 1.07 |
|  |  | (0.99-1.15) | (0.99-1.15) | (0.99-1.15) |
|  | Number of active setts outside but within *x* meters | **p = 0.02** | p = 0.06 | **p = 0.045** |
|  |  | HR: 1.10 | HR: 1.10 | HR: 1.11 |
|  |  | (1.02-1.18) | (1.00-1.19) | (1.01-1.22) |
|  | Density (/km^2^) of active setts total | p = 0.70 | p = 0.74 | p = 0.54 |
|  |  | HR: 1.02 | HR: 1.02 | HR: 1.03 |
|  |  | (0.93-1.10) | (0.92-1.11) | (0.93-1.14) |
|  | Density (/km^2^) of active setts on the land parcels | p = 0.29 | p = 0.29 | p = 0.29 |
|  |  | HR: 0.64 | HR: 0.64 | HR: 0.64 |
|  |  | (0.56-0.72) | (0.56-0.72) | (0.56-0.72) |
|  | Density (/km^2^) of active setts outside but | p = 0.41 | p = 0.55 | p = 0.35 |
|  | within *x* meters | HR: 1.04 | HR: 1.03 | HR: 1.05 |
|  |  | (0.95-1.12) | (0.93-1.12) | (0.94-1.16) |
| **Herd variables ^3^** | Number of cattle herds tested | **p<0.001** | **p<0.001** | **p<0.001** |
|  |  | HR: 1.32 | HR: 1.35 | HR: 1.31 |
|  |  | (1.19-1.46) | (1.20-1.49) | (1.15-1.46) |
|  | Density (/km^2^) of cattle herds  Tested | p = 0.18 | p = 0.06 | p = 0.07 |
|  |  | HR: 1.11 | HR: 1.18 | HR: 1.18 |
|  |  | (0.96-1.26) | (1.00-1.35) | (1.00-1.35) |

A distinction is made for herds for which consent to survey and/or cull was granted between badger-related and sett-related variables on the land parcels belonging to the farm and the buffer area surrounding them.

Hazard ratios (HR) are quoted with their corresponding 95% confidence interval, and for covariates correspond to the change in the risk of a confirmed bTB breakdown following a doubling of the value of the covariate.

^1^ Relate to the badgers culled during the initial proactive cull.

^2^ Relate to the badger setts identified during the initial survey before herds were allocated to their treatment group.

^3^ Relate to herds tested for bTB during the one year prior to the start of the initial proactive cull.

## Multivariable PH models

The badger-related, sett-related and herd-related variables which demonstrated the most significant univariable associations with the risk of confirmed herd breakdowns were retained for multivariable model building (Tables 8 & 9). Models were constructed by backward elimination, starting with a full model with quadratic terms for each non-categorical variable. Variables were eliminated on the basis of their significance in the model as well as their contribution to the variation in the data by means of a likelihood ratio test (LRT) in which twice the difference in log-likelihoods was compared to a Chi-square ($\chi^{2}$) distribution.

Table 8: Multivariable models, using predictors within 1000m of all land parcels, of the time to first confirmed bTB breakdown for RBCT herds during the period under study.

|  | **Survey-only** | | **Proactive** | | **Reactive** | |
| --- | --- | --- | --- | --- | --- | --- |
|  | Model A ^1^ | Model B | Model A | Model B | Model A | Model B |
| Number of *M. bovis* + badgers culled on the land parcels ^2^ | NA | NA | ---- | ---- | NA | NA |
| Number of *M. bovis* + badgers culled outside but within 1000m | NA | NA | p<0.001 | p<0.001 | NA | NA |
|  |  |  | HR: 1.27 | HR: 1.24 |  |  |
|  |  |  | (1.20-1.35) | (1.17-1.32) |  |  |
| Number of active setts on the land parcels ^3^ | ---- | ---- | ---- | ---- | ---- | ---- |
| Number of active setts outside but within 1000m | ---- | p = 0.03 | ---- | ---- | ---- | ---- |
|  |  | HR: 1.11 |  |  |  |  |
|  |  | (1.02 - 1.20) |  |  |  |  |
| Density (/km^2^) of active setts on the land parcels | NA | NA | NA | NA | ---- | ---- |
| Density (/km^2^) of active setts outside but within 1000m | NA | NA | NA | NA | ---- | ---- |
| Number of cattle herds tested ^4^ | p = 0.001 | p = 0.01 | NA | NA | p < 0.001 | p < 0.001 |
|  | HR: 1.33 | HR: 1.26 |  |  | HR: 1.30 | HR: 1.35 |
|  | (1.15 -1.50) | (1.08-1.43) |  |  | (1.15 -1.46) | (1.20 -1.51) |
| Density (/km^2^) of cattle herds tested | NA | NA | ---- | ---- | NA | NA |
| Herd type [DAIRY] | p = 0.29 | p = 0.19 | p = 0.01 | p = 0.02 | p = 0.14 | p = 0.16 |
|  | HR: 1.14 | HR: 1.17 | HR: 1.37 | HR: 1.34 | HR: 0.83 | HR: 0.84 |
|  | (1.00 -1.30) | (0.92-1.50) | (1.07-1.76) | (1.05-1.72) | (0.65 -1.05) | (0.65-1.08) |
| Herd size | p = 0.02 | p = 0.02 | p = 0.02 | p = 0.03 | p = 0.002 | p = 0.001 |
|  | HR: 1.10 | HR: 1.10 | HR: 1.27 | HR: 1.10 | HR: 1.14 | HR: 1.15 |
|  | (1.02-1.18) | (1.02-1.19) | (1.20-1.35) | (1.02-1.19) | (1.06-1.23) | (1.06-1.23) |
| Farm area | p<0.001 | p < 0.001 | p <0.001 | p <0.001 | p < 0.001 | p = 0.001 |
|  | HR: 1.23 | HR: 1.24 | HR: 1.23 | HR: 1.24 | HR: 1.20 | HR: 1.18 |
|  | (1.14-1.33) | (1.14-1.34) | (1.12-1.33) | (1.14-1.35) | (1.10-1.30) | (1.09-1.28) |
| bTB historic incidence within trial area | NA | p = 0.01 | NA | p <0.001 | NA | p < 0.001 |
|  |  | HR: 1.55 |  | HR: 1.84 |  | HR: 1.76 |
|  |  | (1.23-1.87) |  | (1.56-2.11) |  | (1.49-2.03) |

Hazard ratios (HR) are quoted with their corresponding 95% confidence interval, and for covariates correspond to the change in the risk of a confirmed bTB breakdown following a doubling of the value of the covariate.

The --- means that an individual predictor was not significant and removed from the model, while NA corresponds to variables that were not included following the screening process.

^1^ Models are adjusted for herd size, herd type and farm area (model A); herd size, herd type, farm area and bTB historic incidence within the trial area (model B).

^2^ Relate to the badgers culled during the initial proactive cull.

^3^ Relate to the badger setts identified during the initial survey.

^4^ Relate to herds tested for bTB during the one year prior to the start of the initial proactive cull.

Table 9: Multivariable models, using predictors within 1500m of all land parcels, of the time to first confirmed bTB breakdown for RBCT herds during the period under study.

|  | **Survey-only** | | **Proactive** | | **Reactive** | |
| --- | --- | --- | --- | --- | --- | --- |
|  | Model A ^1^ | Model B | Model A | Model B | Model A | Model B |
| Number of *M. bovis* + badgers culled on the land parcels ^2^ | NA | NA | ---- | ---- | NA | NA |
| Number of *M. bovis* + badgers culled outside but within 1500m | NA | NA | p<0.001 | p<0.001 | NA | NA |
|  |  |  | HR: 1.24 | HR: 1.21 |  |  |
|  |  |  | (1.18-1.31) | (1.14-1.28) |  |  |
| Number of active setts on the land parcels ^3^ | ---- | ---- | ---- | ---- | ---- | ---- |
| Number of active setts outside but within 1500m | p = 0.04 | p = 0.02 | ---- | ---- | p = 0.049 | ---- |
|  | HR: 1.11 | HR: 1.13 |  |  | HR: 1.11 |  |
|  | (1.01 - 1.21) | (1.03 - 1.23) |  |  | (1.01 - 1.22) |  |
| Density (/km^2^) of active setts on the land parcels | NA | NA | NA | NA | ---- | ---- |
| Density (/km^2^) of active setts outside but within 1500m | NA | NA | NA | NA | ---- | ---- |
| Number of cattle herds tested ^4^ | p = 0.001 | p = 0.04 | NA | NA | p = 0.01 | p < 0.001 |
|  | HR: 1.30 | HR: 1.22 |  |  | HR: 1.25 | HR: 1.31 |
|  | (1.12 -1.49) | (1.03-1.41) |  |  | (1.09 -1.41) | (1.15 -1.47) |
| Density (/km^2^) of cattle herds tested | NA | NA | ---- | ---- | NA | NA |
| Herd type [DAIRY] | p = 0.30 | p = 0.19 | p = 0.02 | p = 0.03 | p = 0.15 | p = 0.17 |
|  | HR: 1.14 | HR: 1.18 | HR: 1.33 | HR: 1.31 | HR: 0.83 | HR: 0.84 |
|  | (0.90 -1.47) | (0.92-1.74) | (1.04-1.71) | (1.02-1.68) | (0.65 -1.07) | (0.65-1.08) |
| Herd size | p = 0.02 | p = 0.02 | p = 0.01 | p = 0.01 | p < 0.001 | p = 0.001 |
|  | HR: 1.10 | HR: 1.10 | HR: 1.12 | HR: 1.12 | HR: 1.15 | HR: 1.15 |
|  | (1.02-1.18) | (1.02-1.19) | (1.04-1.21) | (1.03-1.20) | (1.07-1.24) | (1.07-1.24) |
| Farm area | p<0.001 | p < 0.001 | p = 0.005 | p <0.001 | p < 0.001 | p = 0.001 |
|  | HR: 1.25 | HR: 1.26 | HR: 1.23 | HR: 1.25 | HR: 1.18 | HR: 1.20 |
|  | (1.15-1.34) | (1.16-1.35) | (1.13-1.33) | (1.15-1.35) | (1.107-1.28) | (1.10-1.30) |
| bTB historic incidence within trial area | NA | p = 0.01 | NA | p <0.001 | NA | p < 0.001 |
|  |  | HR: 1.57 |  | HR: 1.82 |  | HR: 1.75 |
|  |  | (1.25-1.90) |  | (1.55-2.10) |  | (1.48-2.02) |

Hazard ratios (HR) are quoted with their corresponding 95% confidence interval, and for covariates correspond to the change in the risk of a confirmed bTB breakdown following a doubling of the value of the covariate.

The --- means that an individual predictor was not significant and removed from the model, while NA corresponds to variables that were not included following the screening process.

^1^ Models are adjusted for herd size, herd type and farm area (model A); herd size, herd type, farm area and bTB historic incidence within the trial area (model B).

^2^ Relate to the badgers culled during the initial proactive cull.

^3^ Relate to the badger setts identified during the initial survey.

^4^ Relate to herds tested for bTB during the one year prior to the start of the initial proactive cull.

The effects of triplet and historic bTB incidence within the trial areas could be not be simultaneously estimated inside each treatment group as a lack of statistical power, and separate multivariable models were tested adjusting for herd type, herd size, farm area and triplet (Table 10).

Table 10: Alternative multivariable models (adjusted for herd size, herd type, farm area and triplet) of the time to first confirmed bTB breakdown for RBCT herds within a survey-only, proactive or reactive area during the period under study.

|  | **Survey-only** | | | **Proactive** | | | **Reactive** | | |
| --- | --- | --- | --- | --- | --- | --- | --- | --- | --- |
|  | 500m | 1000m | 1500m | 500m | 1000m | 1500m | 500m | 1000m | 1500m |
| Number of *M. bovis* + badgers culled on land parcels ^1^ | NA | NA | NA | ---- | ---- |  | NA | NA | NA |
| Number of *M. bovis* + badgers culled | NA | NA | NA | p < 0.001 | p<0.001 | p<0.001 | NA | NA | NA |
| outside but within *x* m |  |  |  | HR: 1.21 | HR: 1.25 | HR: 1.24 |  |  |  |
|  |  |  |  | (1.11-1.31) | (1.16-1.34) | (1.17-1.53) |  |  |  |
| Number of active setts on the land parcels ^2^ | ---- | ---- | ---- | ---- | ---- | ---- | NA | NA | NA |
| Number of active setts outside but within *x* m | ---- | ---- | ---- | ---- | ---- | ---- | p = 0.03 | ---- | p = 0.03 |
|  |  |  |  |  |  |  | HR: 1.12 |  | HR: 1.19 |
|  |  |  |  |  |  |  | (1.02-1.23) |  | (1.04-1.35) |
| Density (/km^2^) of active setts on the land parcels | NA | NA | NA | ---- | ---- | ---- | NA | NA | NA |
| Density (/km^2^) of active setts outside but within *x* m | NA | NA | NA | ---- | ---- | ---- | NA | NA | NA |
| Number of cattle herds tested ^3^ | p<0.001 | p<0.001 | p<0.001 | NA | NA | NA | p < 0.001 | p < 0.001 | p < 0.001 |
|  | HR: 1.42 | HR: 1.55 | HR: 1.60 |  |  |  | HR: 1.49 | HR: 1.68 | HR: 1.53 |
|  | (1.25 -1.60) | (1.35 -1.74) | (1.4-1.82) |  |  |  | (1.32 -1.66) | (1.48 -1.87) | (1.31 -1.75) |
| Density (/km^2^) of cattle herds tested | NA | NA | NA | ---- | ---- | ---- | NA | NA | NA |
| Herd type [DAIRY] | p = 0.15 | p = 0.13 | p = 0.14 | p = 0.004 | p = 0.003 | p = 0.006 | p = 0.44 | p = 0.42 | p = 0.43 |
|  | HR: 1.20 | HR: 1.21 | HR: 1.21 | HR: 1.46 | HR: 1.49 | HR: 1.44 | HR: 0.90 | HR: 0.90 | HR: 0.90 |
|  | (0.94 -1.55) | (0.94 -1.56) | (0.9 -1.55) | (1.13-1.90) | (1.15-1.93) | (1.11-1.86) | (0.69 -1.17) | (0.70-1.17) | (0.69-1.17) |
| Herd size | p = 0.01 | p = 0.02 | p = 0.02 | p = 0.01 | p = 0.01 | p = 0.004 | p < 0.001 | p < 0.001 | p < 0.001 |
|  | HR: 1.11 | HR: 1.11 | HR: 1.11 | HR: 1.12 | HR: 1.12 | HR: 1.21 | HR: 1.18 | HR: 1.17 | HR: 1.17 |
|  | (1.03-1.20) | (1.03-1.19) | (1.03-1.2) | (1.03-1.21) | (1.03-1.21) | (1.06-1.37) | (1.09-1.27) | (1.109-1.25) | (1.09-1.26) |
| Farm area | p < 0.001 | p < 0.001 | p < 0.001 | p <0.001 | p = 0.001 | p = 0.001 | p = 0.36 | p = 0.07 | p = 0.14 |
|  | HR: 1.21 | HR: 1.22 | HR: 1.22 | HR: 1.21 | HR: 1.31 | HR: 1.31 | HR: 1.05 | HR: 1.10 | HR: 1.09 |
|  | (1.11-1.32) | (1.11-1.32) | (1.12-1.3) | (1.10-1.32) | (1.12-1.52) | (1.12-1.52) | (0.94-1.17) | (1.00-1.21) | (0.98-1.20) |
| triplet | p <0.001 | p <0.001 | p <0.001 | p <0.001 | p <0.001 | p <0.001 | p <0.001 | p <0.001 | p <0.001 |

Hazard ratios (HR) are quoted with their corresponding 95% confidence interval, and for covariates correspond to the change in the risk of a confirmed bTB breakdown following a doubling of the value of the covariate.

The --- means that an individual predictor was not significant and removed from the model, while NA corresponds to variables that were not included following the screening process.

^1^ Relate to the badgers culled during the initial proactive cull.

^2^Relate to the badger setts identified during the initial survey.

^3^ Relate to herds tested for bTB during the one year prior to the start of the initial proactive cull.

## Testing for a correlation between farm area/herd size and the number of “on” movements for survey herds.

Data on survey herds’ movement were not available for the whole duration of the RBCT. However, we extracted movement data from the Cattle Tracing System database for survey herds in 2003 (sufficiently late after the 2001 foot and mouth disease outbreak for normal herd movements to have resumed). Herds registered as belonging to dealers (n = 5) were removed from the dataset. Farm area and the number of “on” movements were correlated (Pearson's product moment coefficient = 0.17, p< 0.001) as were herd size and the number of “on” movements (Pearson's product moment coefficient = 0.13, p< 0.001).

Figure 4: Relationship between farm area (left) and herd size (right) versus the number of “on” movements for survey herds in 2003.


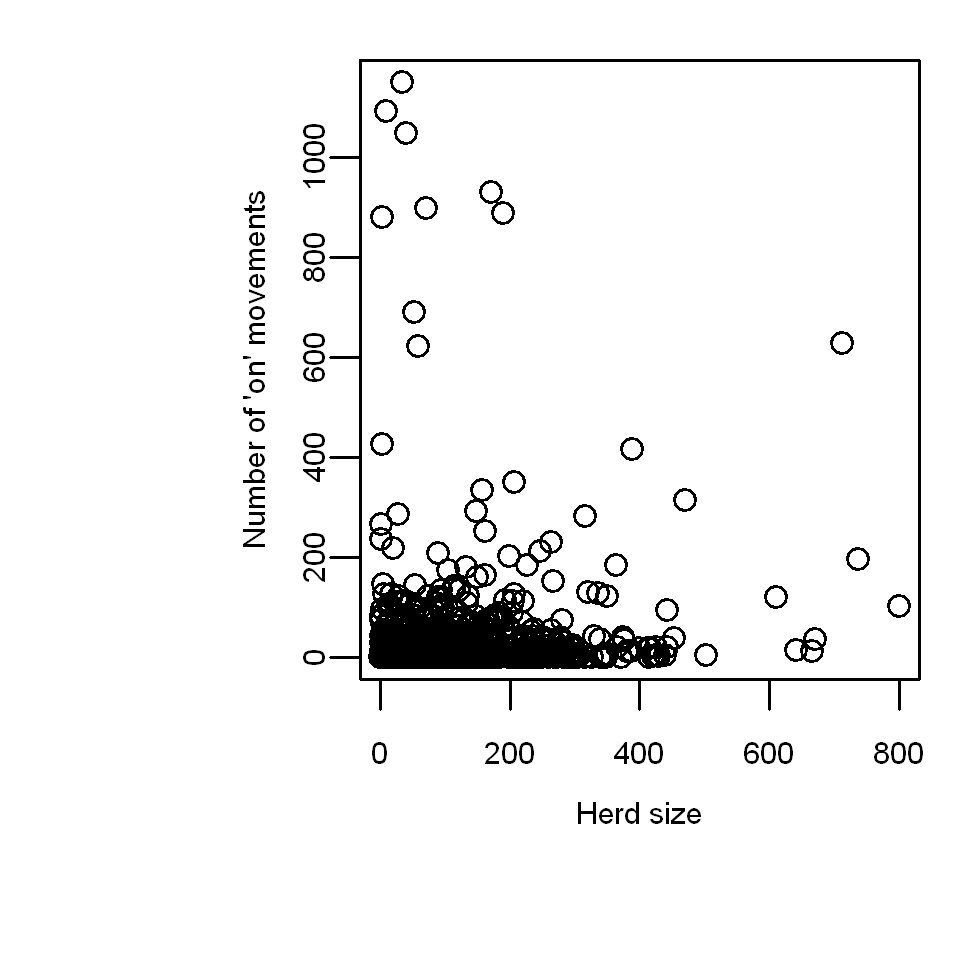

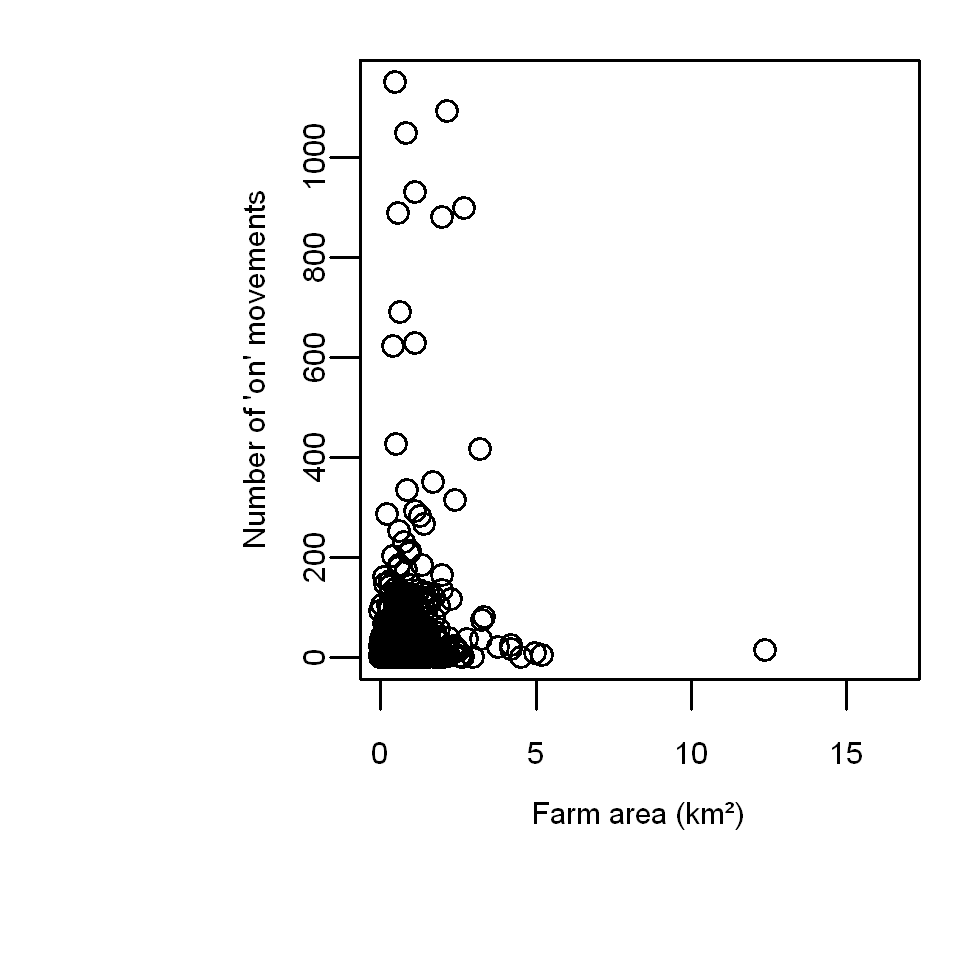


## Testing for a relationship between herd size and herd type.

We found that diary-herds tended to be significantly larger than other herd types (Figure 5) using t-test based on logged herd sizes (p<0.001).

Figure 5: Boxplot of the relationship between herd type and herd size.


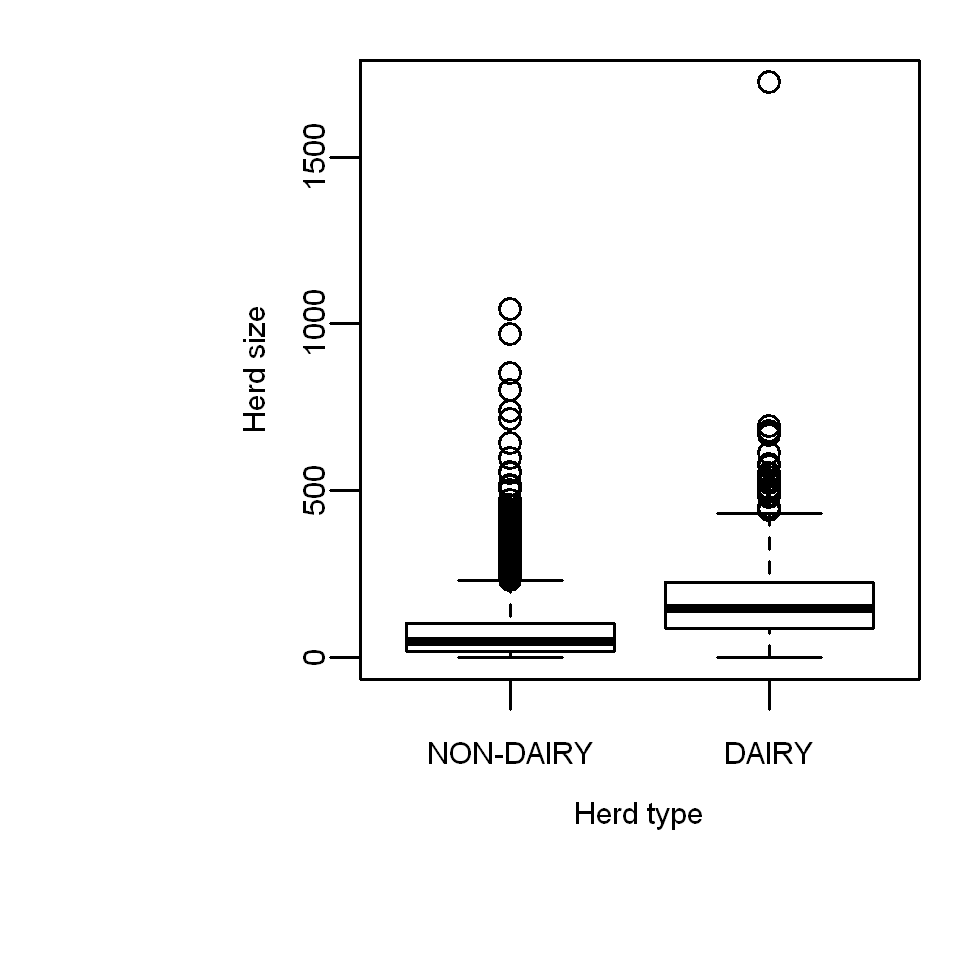

Supplement: Supporting Information S1 — Univariable models and alternative multivariable models of herd-level bTB risk. (DOC) [file pone.0018058.s001.doc]
